# Laser-induced structural modification in calcium aluminosilicate glasses using molecular dynamic simulations

**DOI:** 10.1038/s41598-021-88686-7

**Published:** 2021-05-04

**Authors:** Sean Locker, Sushmit Goyal, Matthew E. McKenzie, S. K. Sundaram, Craig Ungaro

**Affiliations:** 1 Kazuo Inamori School of Engineering, The New York State College of Ceramics, Ultrafast Materials Science and Engineering Laboratory (U-Lab), Alfred University, Alfred, NY 14802 USA; 2 Corning Incorporated, Science and Technology Division, Corning, NY 14831 USA

**Keywords:** Ultrafast photonics, Glasses, Atomistic models

## Abstract

Glass structures of multicomponent oxide systems (CaO–Al_2_O_3_–SiO_2_) are studied using a simulated pulsed laser with molecular dynamics. The short- and intermediate-range order structures revealed a direct correlation between the transformation of Al^(IV)^ to Al^(V)^, regions of increased density following laser processing, inherent reduction in the average T–O–T (T = Al, Si) angle, and associated elongation of the T–O bonding distance. Variable laser pulse energies were simulated across calcium aluminosilicate glasses with high silica content (50–80%) to identify densification trends attributed to composition and laser energy. High-intensity pulsed laser effects on fictive temperature and shockwave promotion are discussed in detail for their role in glass densification. Laser-induced structural changes are found to be highly dependent on pulse energy and glass chemistry.

## Introduction

The application of photo-modified materials in industry reflect the importance in understanding changes to the resolving microstructure. Early studies focused on micromachining surface structures, but quickly evolved into generating 3-dimensional refractive index-modified structures. Femtosecond pulsed laser exposure to materials has become an extensively studied topic^1–7^. Ultrafast pulsed lasers have undergone significant technological breakthroughs, enabling the development and innovation of materials in diverse fields^8–11^. The application of high-intensity laser pulses on transparent materials remains an active topic in large part due to the lack of understanding laser pulse—glass interaction.

Calcium aluminosilicate (CAS) glasses are well studied in materials research because of their superior mechanical- and physical-properties^12–15^. While prior works have investigated the structural dependence on composition and temperature^16–20^, studies of pressure induced structural changes primarily focus on peraluminous (R < 1)^20^ and percalcic (R > 1) glasses^12,19–22^. Limited work probing the silica-rich space along the tectosilicate join (R = CaO/Al_2_O_3_ = 1) has been reported^13–15,23^, and only a handful investigate pressure induced structural changes in the silica-rich CAS along the tectosilicate join^23–25^.

Fused silica has been the primary glass studied in femtosecond laser exposure due to its primitive structure; including experimental and simulated studies^26–28^. Its enhanced thermal–mechanical properties and wide optical transmissivity, extending from the infrared (IR) to ultraviolet (UV), make it an important material used in industry. The densification of fused silica has been documented under extensive experimental conditions such as: hydrostatic compression^29^, neutron irradiation^30,31^, femtosecond pulse laser irradiation^32–34^, and shock-wave propagation^35,36^. Experimental studies in which fused silica was exposed to femtosecond laser pulses, formation of filamentation, ablation, and waveguides have been reported. Chan et al.^1^ have shown a positive correlation between pulse energy (P_E_) and Si–O ring structures from 5- and sixfold to 3- and fourfold coordination. Computer simulations support the attribution of experimental densification in pure silicate glasses to the reduction of Si–O ring sizes^35^. Understanding the effects of glass modifiers added to the system under ambient conditions is pertinent to resolving thermal and pressurized effects.

The addition of Al and Ca into the pure SiO_2_ system creates sites with reduced bond strength, therefore more easily deformed in comparison to fused silica. Aluminum, like it’s silicon counterpart, is integrated into the SiO_x_ structure with tetrahedral geometry, under four-fold coordination. However, it’s prone to form over-coordinated (Al^(V)^) species when exposed to elevated temperature and pressure, enhancing its mechanical properties. Five-coordinated Al, along the tectosilicate join, have been widely reported by Neuville^19,37^, Stebbins^38^ and others^13,23,39^, showing considerable deviation between Al^(V)^ concentrations. Several MD and ab initio molecular dynamics (AIMD) simulated CAS glasses, along the tectosilicate join, report a range of five-coordinated aluminum from 1.4% to approximately 8% in CaAl_2_Si_2_O_8_^13,24,39^. Using ^27^Al Nuclear Magnetic Resonance (NMR), Stebbins^38^ and Neuville^37^ reported a range of Al^(V)^ for slow and fast quenched CaAl_2_Si_2_O_8_ from 7.5–9% and 6.5–7%, respectively. Based on these results, five-coordinated Al in CAS glasses thermally equilibrated to room temperature are highly dependent on potential energies as well as fictive temperature^13,38^. Increased Al-coordination occurs in conjunction with triclustered oxygen (TBO) to compensate for the presence of non-bridging oxygen (NBO). Due to the equal concentration of CaO and Al_2_O_3_, the system has no net charge. In theory, these glasses should be void of any NBO; however, Stebbins et al.^38^ have demonstrated, using ^17^O NMR, the presence of NBO in otherwise charge neutral CAS glasses, also revealing most NBO occur at SiO_4_ sites^12^. Agnello et al.^13^ provide a detailed analysis of the Al^(V)^, in which all were Q^5^ (meaning no NBO bonds were present) and 2-out-of-5 were TBO, while the other three bonds are bridging oxygens (BO). For CAS systems along the tectosilicate join, the number of NBO decreases as silica content increases^16^.

Computer modeling of spatially confined laser-induced processes has largely focused on thermal evolution as a function of time, energy, and pressure. Recent studies have shown the effects of femtosecond pulse laser interaction with metallic surfaces through a two-temperature model (TTM)^9,40^. Applying this model to amorphous materials, like glass, is challenging due to lack of long-range order and covalent bonding. Instead, this study focuses on applying translational kinetic energy in a localized region and allowing the thermal energy to dissipate through the system. Due to experimental limitations, modeling provides a critical insight into highly localized structural gradients and chemistries as a function of glass composition and pulse energy. To the best of our knowledge, this is the first systematic effort of investigating simulated pulse laser-glass interaction with CAS glass and the resolving structural dependence on laser-energy and composition.

The motivation for this research stems from the lack of understanding localized glass structure after pulse laser exposure, and the effects of glass chemistry and pulse energy on the laser-modified region. In this work, we report the results of molecular dynamic (MD) simulations aimed at investigating the effects of composition and laser energy on short and intermediate range order, microstructure, and residual density profiles in silica-rich calcium aluminosilicate (CAS) glasses along the tectosilicate join (CaO/Al_2_O_3_ = 1). We will present the methodology used in simulating both glass formation as well as laser exposure and determine the mechanism responsible for laser induced densification.

## Methods

### Glass formation

Calcium aluminosilicate systems were modeled using Large-scale Atomic/Molecular Massively Parallel Simulator (LAMMPS)^41^. We have chosen the force field developed by Pedone et al.^42^ based on adequate agreement with silicate structural features. The initial structures were created using the correct stoichiometric ratio and experimentally calculated mass densities, seen in Table 1, that produced roughly a 1 million atom sized system of the targeted composition. Each system had an approximate size of 240 × 240 × 240 Å^3^.

**Table 1:** Simulated calcium aluminosilicate glass compositions and respective densities.

Glass code	Composition			Density (g/cm^3^)
	CaO	Al_2_O_3_	SiO_2_	
CAS10.80	10	10	80	2.414
CAS15.70	15	15	70	2.523
CAS20.60	20	20	60	2.672
CAS25.50	25	25	50	2.708

Glasses will further be referred to by the code and pulse energy in subscript format.

**Figure 1:**
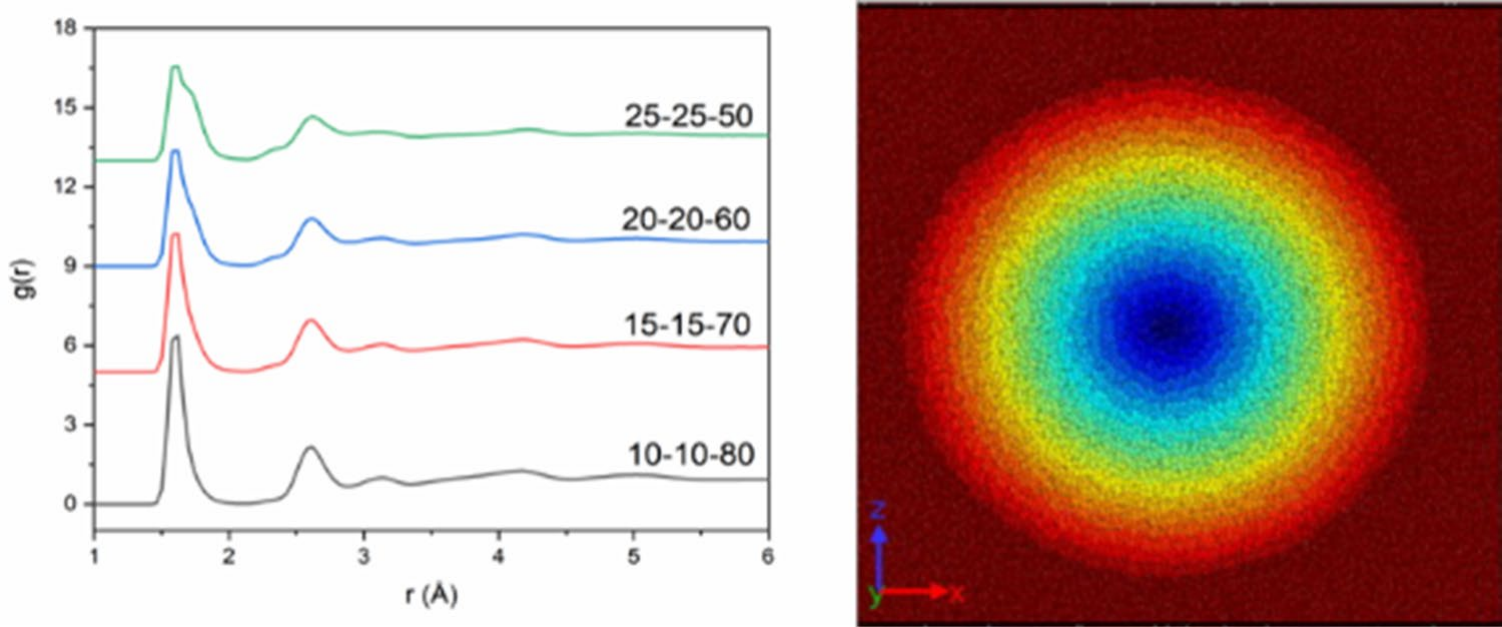
Radial distribution functions for each starting glass composition (left), illustration of the simulated system with assigned shells ranging from 1 to 20 (Ovito, 2.6, https://www.ovito.org/) (right).

Long-range Coulombic interaction were represented by a particle–particle particle mesh (PPPM) solver at an accuracy of 10^–4^ and a timestep of 2 fs. The system was heated to a temperature of 4000 K, allowing the atoms to move for 50 ps. The randomized structure was quenched to 300 K over 0.5 ns, using the NVT ensemble. The simulated glass was finally relaxed to atmospheric pressure under a controlled pressure and temperature ensemble for 1 ns using a Berendsen thermostat and Berendsen barostat^43^. Quench rates of 3.7 and 0.74 K/ps were also tested to ensure little to no change in defect concentration. Radial distribution functions (g(r)) for initial configurations are shown in Fig. 1. The visual software OVITO^44^ was used to investigate microstructure and atom distribution of simulated glasses. To calculate structural characteristics, using Visual Molecular dynamics^45^ (VMD), cutoff distances were chosen based on the first minimum of the pair radial distribution function (PRDF): Si–O, Al–O, Ca–O cutoff distances of 2.15, 2.25 and 3.15 Å, respectively. Running coordination numbers for Al–Al, Si–Si and Al-Si were calculated by integrating the radial distribution functions, using a delta of 0.005.

### Laser exposure

Simulating laser exposure is applied through a heat flux, *J*, across a defined region which we will be refer to as the hotspot or focal region.

1 $$J = \frac{1}{V}\left[ {\mathop \sum \limits_{i} e_{i} {\varvec{v}}_{i} - \mathop \sum \limits_{i} S_{i} v_{i} } \right]$$

2 $$J = \frac{1}{V}\left[ {\mathop \sum \limits_{i} e_{i} {\varvec{v}}_{i} - \mathop \sum \limits_{i < j} (f_{ij} \bullet v_{j} )x_{ij} } \right]$$

3 $$J = \frac{1}{V}\left[ {\mathop \sum \limits_{i} e_{i} {\varvec{v}}_{i} + \frac{1}{2}\mathop \sum \limits_{i < j} (f_{ij} \bullet (v_{i} + v_{j} ))x_{ij} } \right]$$

The first term in the equation for *J* is the per-atom kinetic and potential energy (*e*_*i*_), *S*_*i*_ in the second term represents the calculated per-atom stress tensor, volume (V) and *v*_*i*_ is a 3 × 3 matrix–vector multiplier. The laser source is applied at (x,y) = (116.5, 116.5) nm along the entire z-axis. Simulated laser heat is applied to this system in the form of a gaussian profile using the form of a normal distribution,

$$y = \frac{A}{{\sigma \sqrt {2\pi } }}e^{{ - \frac{{\left( {x - \mu } \right)^{2} }}{{2\sigma^{2} }}}}$$

where $\sigma$ is half pulse width divided by root two, A is number of atoms within the heated region multiplied by energy/atom (method for calculating this energy will be discussed later), *x* is step number multiplied by the timestep (2 fs), and *μ* is the pulse width (10 ps). During the laser exposure process, a Nose–Hoover global thermostat was applied outside of the heated region to simulate thermal dissipation from the laser pulse^46^. A total of 80 ps trajectory was conducted to ensure thermal equilibration. To maximize laser modified volume, the simulated focal point was elongated to a cylinder. As previously discussed, the structural modifications fluctuate across the length of the box, therefore we cylindrically chunk along the z-axis with a shell width of 5 nm, seen in Fig. 1. Although there are 48 chunks in the entire system, we will show perturbations are only observed in the first twenty shells. The remainder of this paper we will refer to specific shell numbers or a series of shells when referring to the modified system. For example, shell number 5 would be a shell starting from 25 nm and ending 30 nm from the center of the periodic box.

To calculate per atom energy, we used an in-house script that with inputs from experimental laser parameters (pulse width, peak intensity, repetition rate, and spot size) and material properties to model nonlinear laser absorption during experimentation. In this work, per atom energy represents the transfer of energy between the electronic and ionic systems while the experimental process (discussed later) involves phonon-electron coupling; both processes yielding similar results. A model similar to that of Couairon et al.^47^ was used, taking into account multiphoton and avalanche absorption, plasma defocusing, and Raman scattering. The amount of energy absorbed per atom in the center of the focal volume due to a Gaussian beam of pulse width of 10 ps and wavelength of 1064 nm focused by a lens of numerical aperture = 0.6 was modeled. Pulse energies of 4.5 uJ, 3.5 uJ, 2.9 uJ, 2.4 uJ, and 2.2 uJ were found to correspond to 100, 50, 25, 15 and 10 eV/atom respectively. Note that both the volume of the absorption region and the percentage of the beam absorbed vary with laser P_E_, resulting in a varying sensitivity to P_E_. The material parameters in the model were for fused silica which may cause some variance in the results; however, this range of energies was chosen to simulate common laser processing conditions. Energy per atom and spot size were optimized to reach target temperatures. A focal volume of less than 5% of the total volume is used to mitigate boundary effects.

## Results and discussion

### Residual density profile

**Figure 2:**
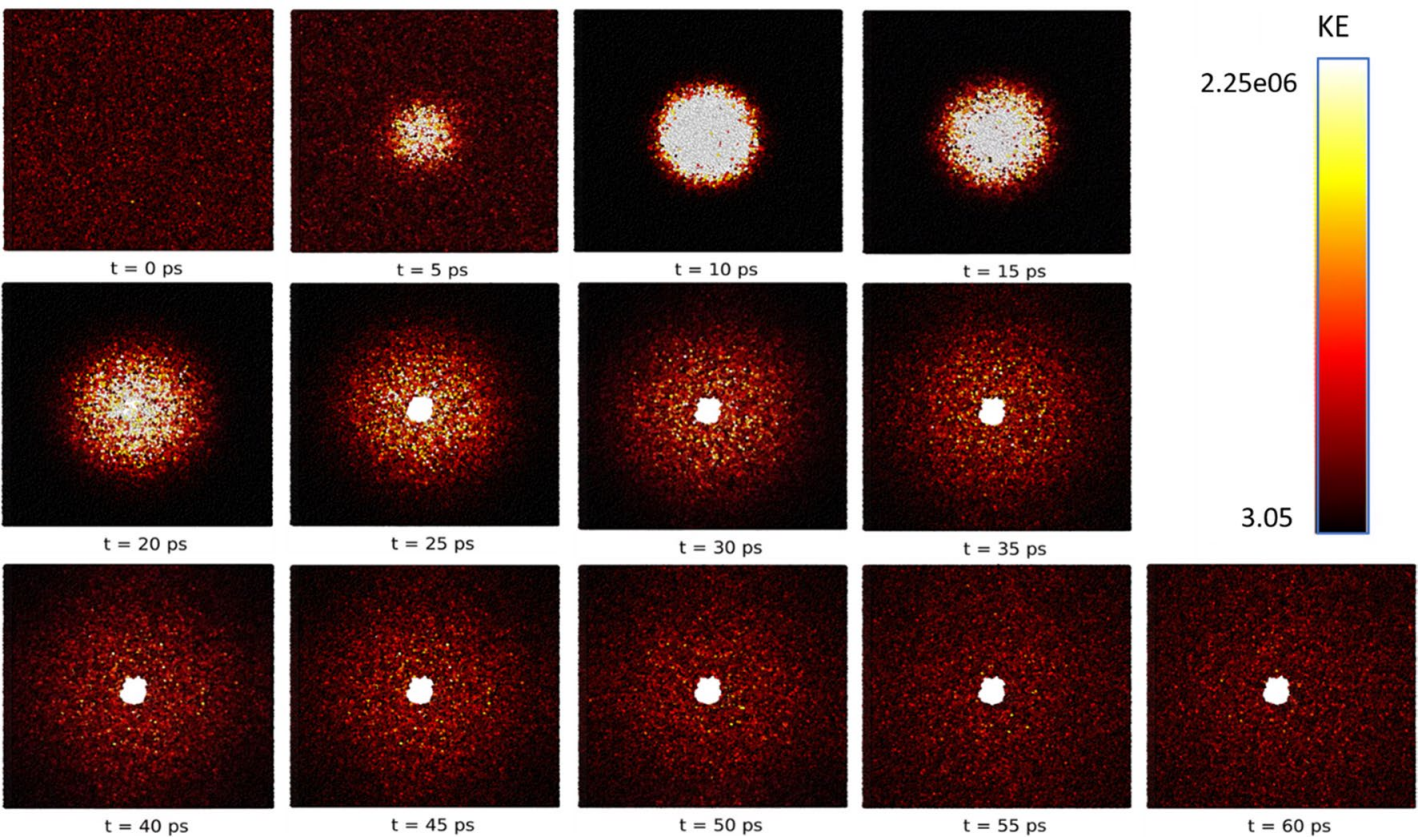
Time-lapse of simulated kinetic energy(kg m^2^ s^−2^) during laser pulse interaction (PE = 100 eV/atom) with calcium aluminosilicate glass (Ovito, 2.6, https://www.ovito.org/).

Figure 2 shows a time-lapse of the simulated laser pulse interaction with CAS10.80_100_. Peak laser fluence occurs at the 10 ps timestep, after which thermal energy dissipates until it’s equilibrated to room temperature. High intensity pulse interaction induces void formation, commonly seen in experimental studies of silicate glasses^6,7,48,49^. Voids were observed in all compositions when the P_E_ ≥ 15 eV/atom. While the pulse reaches significantly higher temperature than the glass, this is because not all energy is translated via heating the system (e.g. fluorescence or plasma-induced shockwave). Formation of voids is accompanied by compaction in the surrounding region.

The modified glass structure was first analyzed by mapping residual density profiles. Changes in density, and concurrent linear refractive index, align with Bhardwaj et al.^50^ results for aluminosilicate and alkali-aluminosilicate glass density after pulse laser exposure. Laser-induced refractive index gradients are the foundation for writing waveguides, and are reported to range 10^–4^ to 10^–2^ in fused silica^2^. These structural modifications, however, are highly dependent on glass composition and laser conditions.

**Figure 3:**
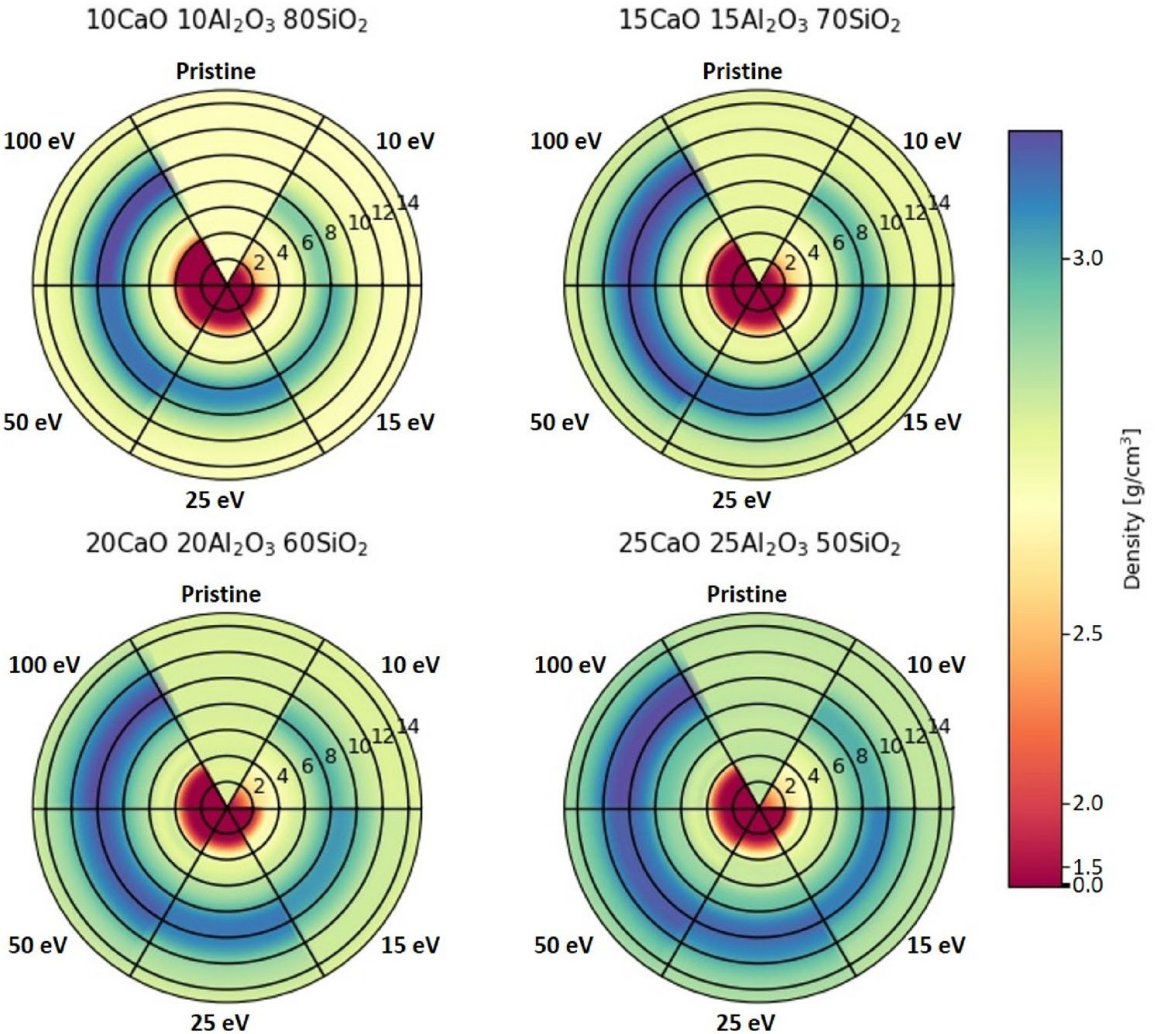
Radial heatmaps of simulated calcium aluminosilicate glass density in pristine condition and after exposure to various simulated pulse energies; inset even numbers denote shells.

Radial heatmaps, in Fig. 3, illustrate per shell density of the pristine and post-laser modified glasses. Simulated initial glass densities show good agreement with experimental values. Density within the focal volume reduces significantly due to void formation. This process occurs in experiments when tightly focused laser pulse energy is absorbed by electrons, producing an electron–ion plasma^6^. Electrons collisionally heat ions within the focal volume, and upon expansion significant pressures build and generate a pressure wave, or shockwave, resulting in artifacts such as voids, change in chemical species and density profiles. As anticipated, higher energies increase void size. Our results are in good agreement with previous studies investigating the effect of P_E_ on void size in fused silica, borosilicate, and aluminosilicate glasses^51^. These studies demonstrate picosecond and femtosecond timescales produce similar trends in void size as a function of P_E_. The observed trend of increasing plasma formation, increasing void size, imposes a greater densification (likewise higher dielectric), strain, and subsequent pressure wave generation^34^.

**Figure 4:**
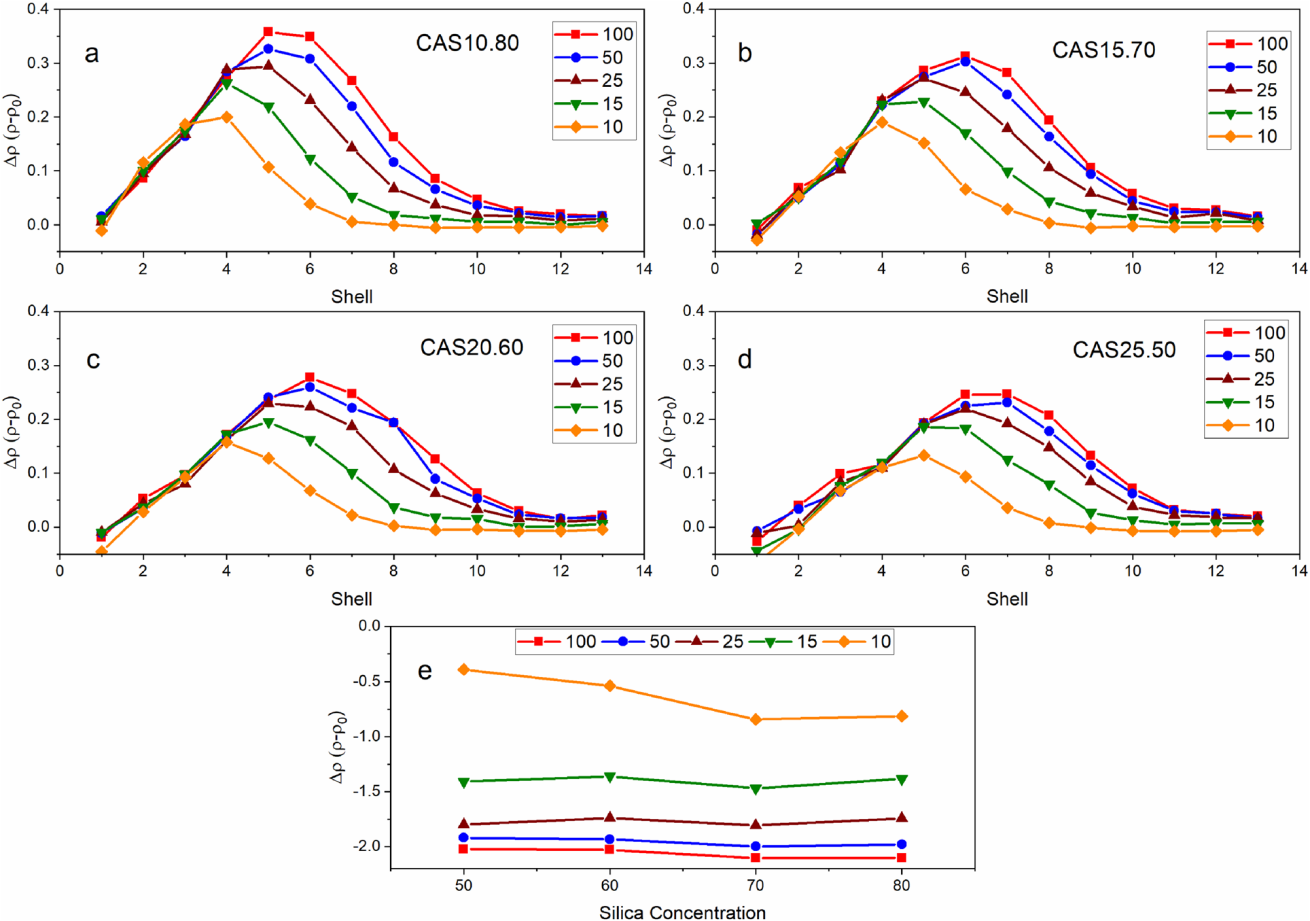
Change in density after simulated laser exposure for shells 1–13 (30–90 Å) in (**a**) CAS10.80, (**b**) CAS15.70, (**c**) CAS20.60, (**d**) CAS25.50 and (**e**) within the focal volume (0–25 Å). Error is within 1%.

Compositional dependence of localized residual density fields for each laser condition are shown in Table 2. Note the trend in single shell maximum density change—the addition of calcium and aluminum reduces the maximum change in density observed in a single shell. Considering the kinetic fragility index, which characterizes how rapidly the relaxation process occurs, we anticipate fragility to increase with larger initial densities^52^. As a result, glasses with high silica concentration (and lower initial density) are more difficult to relax^53^. Highly localized heating from the pulse-glass interaction produces the largest distribution in density profiles for these glasses. Similarly, we see a compositional dependence on the void diameter and change in density within the focal volume presented in Fig. 4 and Table 2. When x = 0.15, 0.20 and 0.25, focal volume density increases 4.2%, 10.8% and 13.5%, respectively, in comparison to CA10.80_100_. The addition of calcium creates NBO at either Si–O or Al-O sites, weakening the tetrahedral network. The reduction in network connectivity reduces the transition temperature (T_g_) and viscosity, therefore increasing the observable viscous flow of the heated glass after removing the simulated pulse^5^. Lower viscosity provides more time for the glassy network to rearrange, confirmed based upon the direct correlation between density and fragility, causing the void to inherently collapse inward, reducing the diameter.

**Table 2:** Simulated localized density changes as a function of composition and laser energy for the shell with the highest change in density, density within the focal point (shell 1–5), and the change in density from shell 1–11.

Composition	P_E_ (eV)	Initial density (g/cm^3^)	Max Δ*ρ* (%)	Focal volume Δ*ρ* (shell 1*–*5)(%)	Overall Δ*ρ* (shell 6–11) (%)
10CaO 10Al_2_O_3_ 80SiO_2_	100	2.57	14.0	− 81.6	8.1
	50	2.57	12.4	− 76.8	7.7
	25	2.57	11.3	− 67.5	7.0
	15	2.57	9.7	− 53.6	5.7
	10	2.57	7.4	− 31.5	4.1
15CaO 15Al_2_O_3_ 70SiO_2_	100	2.68	11.6	− 78.2	6.1
	50	2.68	11.2	− 74.2	5.8
	25	2.68	10.1	− 67.2	5.5
	15	2.68	8.6	− 54.6	4.9
	10	2.68	6.7	− 31.2	3.4
20CaO 20Al_2_O_3_ 60SiO_2_	100	2.78	10.1	− 72.8	4.9
	50	2.78	9.5	− 69.4	4.7
	25	2.78	8.3	− 62.3	4.3
	15	2.78	6.8	− 48.9	3.9
	10	2.78	5.7	− 19.3	2.6
25CaO 25Al_2_O_3_ 50SiO_2_	100	2.86	8.7	− 70.6	4.1
	50	2.86	8.0	− 66.9	3.7
	25	2.86	7.7	− 62.7	3.4
	15	2.86	6.6	− 49.0	3.2
	10	2.86	4.9	− 13.5	2.2

These measurements were average over ten timesteps and standard error less than 1%.

Figure 4 shows the change in density on a per shell basis, for each composition under all simulated laser-exposure conditions (Fig. 4a–d). Density change (Δρ) within the focal volume (diameter of 25 Å), seen in Fig. 4e, as a function of silica concentration and laser energy support the observation that modifier content and void size are inversely proportional. Focal volume Δρ, found in Table 2, varies 11% between CAS10.80 and CAS25.50 with P_E_ = 100 µJ. Nearly every condition we find Δρ for shell 1 (25–30 Å from the origin) synonymous to the pristine glass, CAS25.50 shows slight deviates from this trend. Subsequent shells (representing 5 Å rings) show positive Δρ and maximum densified shell that is dependent on silica content and pulse energy. The maximum observed densification occurs in CAS10.80 (14%). Integrating the function, we calculate area under the curve and find total densification increases with silica concentration. As previously mentioned, glasses with higher silica content have lower fragility and therefore experience enhanced peak densification. The position of peak densification for all conditions support relaxation based on structural rigidity. Based on the overall Δρ between shells 6–11, densification trend becomes asymptotic as P_E_ increases. Further extrapolation to higher energies would likely yield a plateau in densification. Experimental studies in laser exposure of soda-lime silicates (SLS)^40,41^ observe similar behavior in densification as a function of P_E_. Laser-induced densification gradually reduces at increased distance from the focal point; shell 13 (90 Å from the focal point) the density returns to its unmodified condition.

Based on simulated and experimental initial glass densities, we find the magnitude of laser-induced densification is dependent on the system’s free volume. Replacing Al and Ca for Si increases the structure’s packing density, therefore diminishing laser-exposure distortions to the glassy network. The position of peak densification occurring at greater distances from the focal point is attributed to a combination of enhanced fragility and reduced rigidity. Both factor into shockwave propagation during exposure and structural relaxation after the pulse is removed. Additionally, our results indicate a limit to P_E_ effect on densification in area surrounding the focal point.

### Coordination number distribution

**Figure 5:**
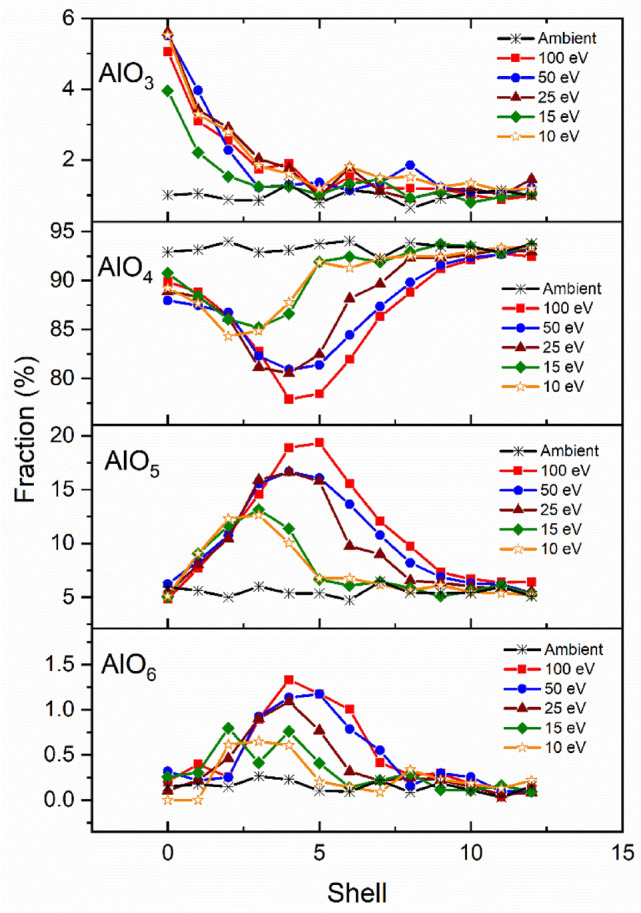
Distribution of CAS10.80 AlO_x_ units (at x = 3, 4, 5 and 6) as a function of shell number. Shell zero represents the combine statistics within the focal volume (shells one through five). These values were averaged over ten timesteps and were all within 2%.

Figure 5 shows the average coordination number distribution per shell of Al species in CAS10.80. The initial condition, shell zero, represents the total coordination within the focal volume (shells one through five). Pressure of the overall system at peak intensity of the simulated pulse was 6.1, 4.5, 2.9, 2.0 and 1.3 GPa for 100, 50, 25, 15 and 10 eV, respectively. Aluminum is predominately four coordinated in ambient conditions for all tectosilicate simulated systems^20^. The unmodified structure has predominantly four- and some five-fold Al species (CAS10.80: 94% and 5%, CAS15.70: 91% and 8%, CAS20.60: 89% and 9%, CAS25.50: 87% and 12%, Al^(IV)^ and Al^(V)^, respectively). Average aluminum coordination changes for CAS15.70, CAS20.60, and CAS25.50 can be found in the supplementary data.

The concentration of AlO_3_ in the unmodified system is ≤ 1% for all compositions and decreases with SiO_2_. These results are in agreement with Agnello^13^ and Tandia^39^, who modeled identical compositions. Hong et al.^12^ show that increasing pressure in percalcic CAS glasses reduces the fraction of AlO_3_ while increasing 4- and fivefold coordination. Based on shockwave propagation and direct relation to SiO_2_ concentration, it seems the increased Al^(III)^ species within the focal volume is attributed to the tetrahedral substitution of Al^3+^ for Si^4+^.

The trend between P_E_ and coordination is consistent across all compositions, while Al^(V)^ concentrations increased with Al_2_O_3_/SiO_2_ ratio. Our results show that the presence of Al^(V)^ in the glass structure are consistent with the observed increase in glass density (Fig. 3a). Previous studies^53^ demonstrated an increase in Al-coordination as a function of hardness and densification. Based on the residual density profiles, in Fig. 4, and the Al^(V)^ coordination in Fig. 5, it is likely the increased coordination state plays a dominant role in the densification and crosslinking of the glass network. Amplified Al(V) species appear inversely proportional to the change in Al^(IV)^. This change is largely attributed to the ability of Al to go from fourfold to fivefold under increased temperature and pressure^35,54^. The unmodified structure has predominantly four- and some five-fold Al species (CAS10.80: 94% and 5%, CAS15.70: 91% and 8%, CAS20.60: 89% and 9%, CAS25.50: 87% and 12%, Al^(IV)^ and Al^(V)^, respectively).

The fraction of AlO_6_ is less than 1% in all compositions in ambient conditions. Simulated P_E_ = 100 eV increased Al^(VI)^ concentration range from 1 to 4% for CAS10.80 and CAS25.50, respectively. Stebbins et al.^21^ show that annealing quenched glasses and lowering their fictive temperatures significantly reduces the population of high-coordinated Al species. Denser silica glasses are formed at fast quench rates and large fictive temperatures^7^. These findings are consistent with our results where the annealed starting structure has near zero Al^(VI)^ concentration.

It is worth noting that the simulated laser-pulses induced a pressure-wave which increases the number density by approximately 12.4% in shells with the highest Al^(V)^ and Al^(VI)^ content. Although the increase in number density is not attributed the larger population of over-coordinated Al, we find that pulse energy and high-coordination are directly related, similar to Stebbins et al.^21^ use of high-resolution^27^Al and ^17^O NMR to show that the content of five-coordinated aluminum increases with fictive temperature.

**Figure 6:**
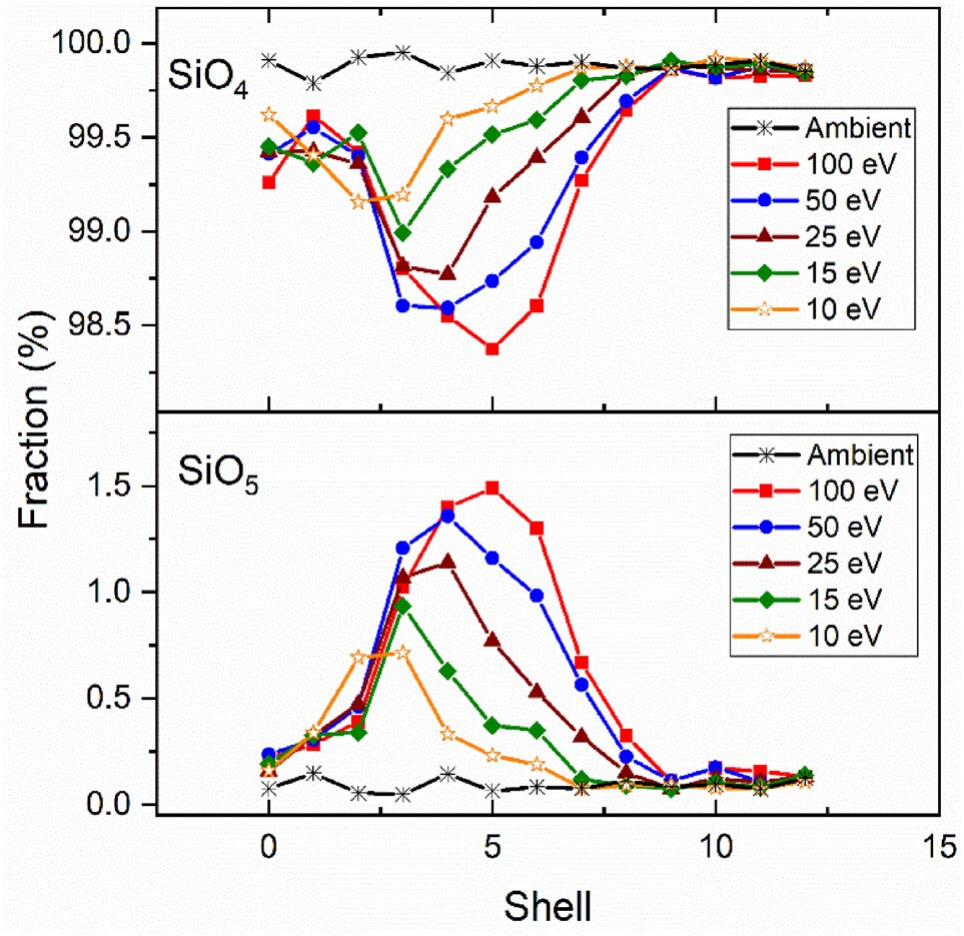
Distribution of CAS10.80 SiO_X_ units (X = 4 and 5) as a function of shell number. Shell zero represents the combine statistics within the focal volume (shells one through five). These values were averaged over ten timesteps and were all within 2%.

Under ambient conditions the coordination number distribution of nearly all Si atoms is four-fold, forming SiO_4_ tetrahedra. The distributions of Si^(IV)^ and Si^(V)^, seen in Fig. 6, follow a similar trend as Al species where the density in regions surrounding voids increases due to the increased fictive temperature and pressure. As Stebbins^38^ and Lee^54^ highlight in aluminosilicates 5- and 6-coordinated Al form much more readily at high temperatures and pressures than Si^(V)^. Previous studies^12,23,54^ show that the Si transition from four to five-fold coordination occurs at pressures ranging from 10 to 15 GPa, all the way up to 40 GPa; however, the maximum pressure predicted in the simulated laser pulse was 6.1 GPa. The densification process around silica tetrahedron primarily result from a reduction in the inter-tetrahedral angle (Si–O–Si), small or undetectable change of interatomic distances, and gradual increase of average atomic coordination^29^. Although our model generates pressures significantly lower than studies observing pressure induced over-coordinated Si (Si^(V)^), Stebbins et al.^55^ have shown its abundance also increases with fictive temperature. The simulated fraction of Si^(V)^ in this study is small and may be unobservable using ^29^Si NMR—further experimental investigation should follow. The highest concentration of laser induced-five-coordinated silicon occurs in CAS10.80. Due to Si^(V)^ dependence on silicon content, we anticipate lower concentrations in CAS15.70, CAS20.60 and CAS25.50. For this reason, our model shows silicon only exists as four-fold in those systems.

### Interatomic bonding

#### Short range order

**Figure 7:**
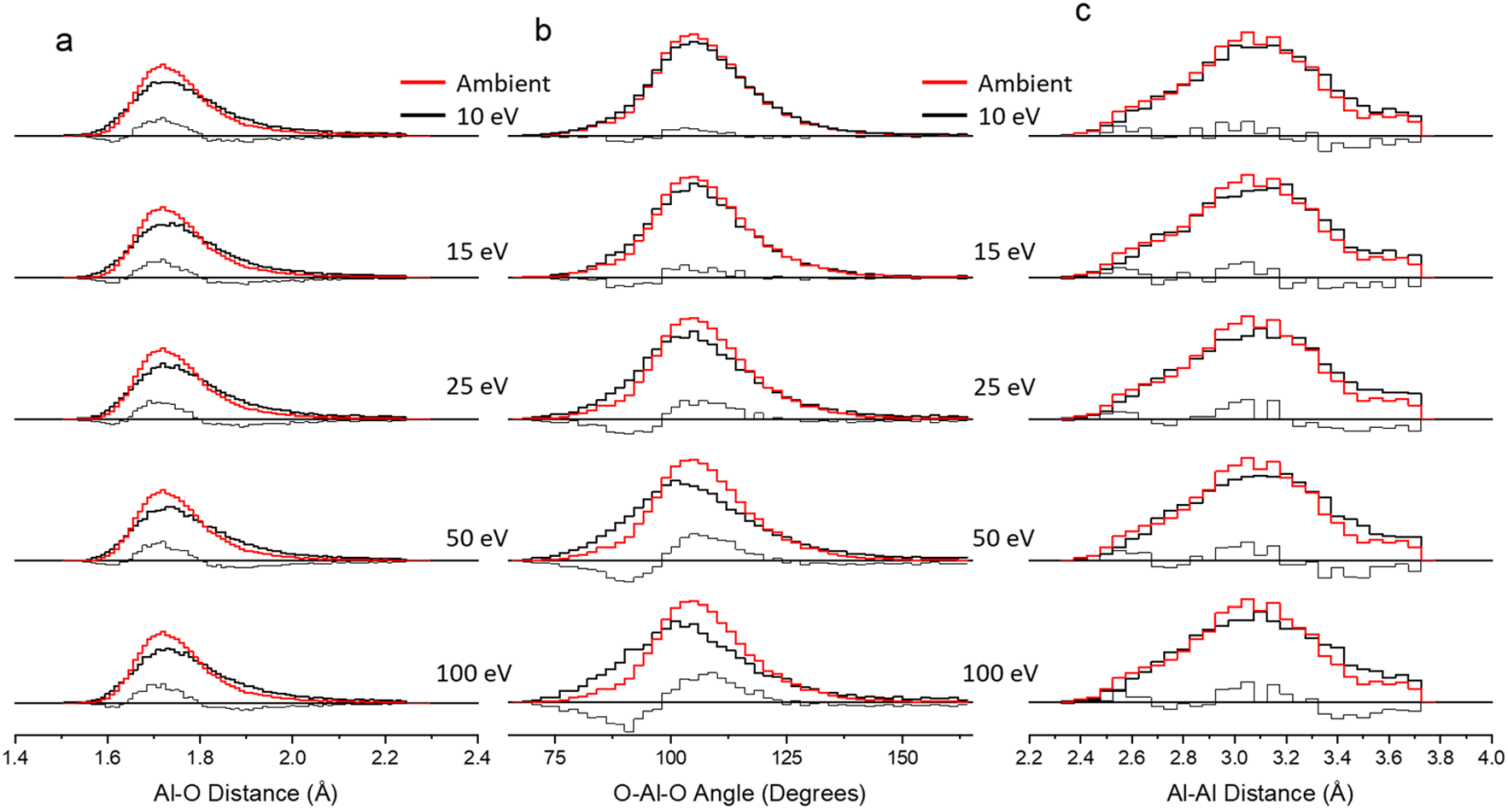
Histogram of (**a**) Al–O distance, (**b**) O–Al–O bond angle, and (**c**) Al–Al distance for 10CaO 10Al_2_O_3_ 80SiO_2_ glass (shells 1–11) after simulating various pulse energy exposures (wide black line) and ambient structure (wide red line). The thin black line under each distribution shows the difference in distributions for visual assistance. Error within 1%.

**Figure 8:**
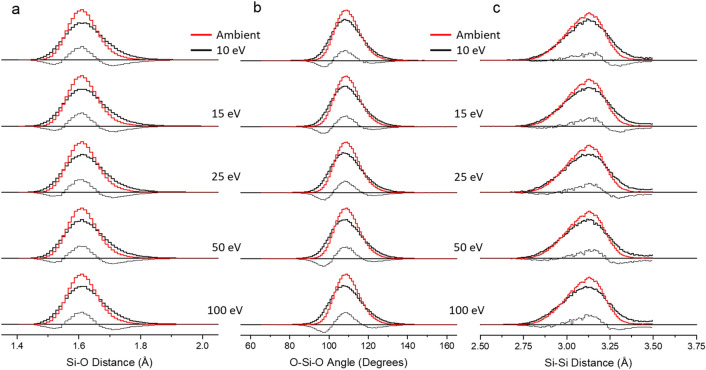
Histogram of (**a**) Si–O distance, (**b**) O–Si–O bond angle, and (**c**) Si–Si distance for 10CaO 10Al_2_O_3_ 80SiO_2_ glass (shells 1–11) after simulating various pulse energy exposures (wide black line) and ambient structure (wide red line). The thin black line under each distribution shows the difference in distributions to help illustrate the differences. Error is within 1%.

Simulated aluminum and silicon interatomic bonding distance distributions over the first eleven shells (55 Å)are shown in Figs. 7 and 8 (normalized y-axis) along with distribution statistics in Tables 3 and 4. To better understand the effect of pulse energy on localized structure, the topology of TO units (T = Al, Si) has been investigated via T–O and T–T radial distribution and O–T–O bond angle distributions; ambient conditions are plotted over each simulated laser condition, including the difference in distributions. Histogramed data and statistics for the other compositions can be found in the suplementary information. Distortions in the glass random network produce a distribution around the ideal bonding angle and interatomic bond distance. We show the effects pulse energy and composition have on the resolving distributions.

**Table 3:** Distribution statistics for Al–O, O–Al–O, and Al–Al under ambient and laser modified conditions in CAS10.80.

Parameter	Ambient	10 eV	15 eV	25 eV	50 eV	100 eV
**Al–O–Al**						
Mean	116.1 ± 0.25	115.8 ± 0.24	115.8 ± 0.25	115.2 ± 0.25	115.7 ± 0.25	115.3 ± 0.25
Median	114.5	113.5	113.2	113.1	113.8	113
Std. dev.	20.5	20.6	20.8	20.4	20.3	20.7
**Al–O–Si**						
Mean	136.8 ± 0.09	135.6 ± 0.09	135.3 ± 0.09	134.6 ± 0.10	134.2 ± 0.09	134.3 ± 0.10
Median	135.7	134.8	134.3	133.6	133	133
Std. dev.	17.9	18.7	18.8	18.9	19	19
**O–Al–O**						
Mean	108.8 ± 0.13	108.6 ± 0.18	108.5 ± 0.14	108.4 ± 0.15	108.3 ± 0.16	108.3 ± 0.16
Median	107.5	106.7	106.4	106.1	105.8	105.7
Std. dev.	13.6	15.8	16.2	16.9	17.2	17.5
**d(Al–O)**						
Mean	1.76 ± 0.00	1.78 ± 0.00	1.78 ± 0.00	1.79 ± 0.00	1.79 ± 0.00	1.80 ± 0.00
Median	1.74	1.76	1.76	1.76	1.77	1.77
Std. dev.	0.01	0.12	0.12	0.12	0.12	0.12
**d(Al–Al)**						
Mean	3.09 ± 0.00	3.12 ± 0.00	3.12 ± 0.00	3.12 ± 0.00	3.13 ± 0.00	3.13 ± 0.00
Median	3.09	3.11	3.12	3.12	3.13	3.13
Std dev.	0.26	0.27	0.27	0.28	0.27	0.28

These values were averaged over ten timesteps and indicate standard error.

**Table 4:** Distribution statistics for Si-O, O-Si-O, and Si-Si under ambient and laser modified conditions in CAS10.80.

Parameter	Ambient	10 eV	15 eV	25 eV	50 eV	100 eV
**Si–O–Si**						
Mean	148.4 ± 0.05	147.3 ± 0.06	146.8 ± 0.06	146.5 ± 0.06	146.2 ± 0.06	146.0 ± 0.06
Median	148.2	147.2	146.7	146.4	146.0	145.8
SD	13.9	14.5	14.8	14.9	15.0	15.0
**O–Si–O**						
Mean	109.3 ± 0.01	109.2 ± 0.02	109.2 ± 0.02	109.2 ± .02	109.2 ± .02	109.2 ± 0.02
Median	108.9	108.6	108.6	108.5	108.4	108.4
SD	6.70	8.30	8.54	8.82	9.01	9.05
**d(Si–O)**						
Mean	1.61 ± 0.00	1.62 ± 0.00	1.62 ± 0.00	1.62 ± 0.00	1.62 ± 0.00	1.62 ± 0.00
Median	1.61	1.62	1.62	1.62	1.62	1.62
SD	0.05	0.07	0.07	0.07	0.07	0.07
**d(Si–Si)**						
Mean	3.098 ± 0.000	3.106 ± 0.001	3.105 ± 0.001	3.105 ± 0.001	3.106 ± 0.001	3.108 ± 0.001
Median	3.102	3.111	3.108	3.108	3.106	3.106
SD	0.110	0.130	0.134	0.135	0.136	0.138

These values were averaged over ten timesteps and indicate standard error.

Four-coordinated Al and Si have a tetrahedral geometry and average bond angle of approximately 109.5°; tetrahedron geometry is comprised of four 109.5° angles, trigonal bipyramidal geometry has three 120°, two 90° and one 180° angle. The average O–Al–O bond angle in CAS10.80 is approximately 108.8° and average Al–O and Al–Al bonding distance of 1.76 and 3.09 Å, respectively. Average Al–O and Al–Al distances in the ambient system (CAS10.80) were 1.76 and 3.09 Å. These values are in agreement with previous simulation^12,17,56–59^ and experimental^20,60,61^ studies. Compositions CAS15.70, CAS20.60 and CAS25.50, under ambient conditions, have an average Al-O bond distance of 1.77 Å and Al-Al distance of 3.10, 3.11 and 3.12 Å, respectively. Minor elongation of the average bond length can be attributed to elevated population in high-coordinated polyhedra. Average O–Al–O bond angle is consistent across all compositions.

Bonding environment for Si under ambient conditions is extremely stable primarily due to its high bond energy^62^. The stability was previously eluded to in the discussion of nearly 100% tetrahedral geometry in high-silica content glasses under extreme conditions; similar findings have also been reported in experiments and simulations^12,20,57,59,63^. Average O–Si–O bond angle of 109.3° is constant in all simulated systems under ambient conditions. Likewise, the calculated Si–O and Si–Si bonding distance of 1.61 and 3.098 Å in agreement with experimental findings^60,61,64^.

Pulsed laser-glass interacion increases the systems disorder through broadening of the O–Al–O and O–Si–O anglular distributions. The standard deviation for both distributions increase with pulse energy (Tables 3 and 4). Although both angle distributions are mostly-symmetric under ambient conditions, laser exposure causes some shift and asymmetry of the peak(unmodified O–Al–O and O–Si–O are centered at 106.5° and 109°, respectively). The O–Al–O bond angle is more susceptible to distortions; P_E_ = 10 and 15 eV show slight reduction around the average angle and increase around 90°, P_E_ = 25, 50, and 100 eV show similar trend with 100 eV distirbution centered at 103° and average of 108.3°; the data reflects a positive skew. Bond angle distribution for O–Si–O shows no change in average bond angle; however, as pulse energy increases the distribution shifts to lower angles with P_E_ = 100 eV centered at 107°. Increasing Al_2_O_3_/SiO_2_ ratio did not have a significant effect on O–Al–O peak position, where bond angle distribution (P_E_ = 100 eV) in CAS15.70, CAS20.60 and CAS25.50 are centered at 102.5°, 102.5°, and 103°. However, it is important to note that enhancing the ratio increases the average O–Al–O distortions when compared to the ambient system.

Laser induced changes to interatomic bonding distance echo the bond angle data, where strucutral modifications are most prominent in Al species and the average Al-O bond length increases with succesively higher pulse energy. Because bond length and interbonding-angle are inversely proportional, the densification process reduces the average bond angle and increases bond length. At P_E_ = 100 eV, Al–O bond length in CAS10.80 increased 0.04 Å, while the average bond distance in compositions CAS15.70, CAS20.60 and CAS25.50 show a slight reduction to 0.03 Å. Calculated increase in Al-O bonding distance is due to increased Al-coordiantion number^12,65^; these findings are consistent with empirical observations in ionic crystals^66^. It is also worth noting the averge Al-O bond length is dependent on temperature and pressure, based on the incremental trend towards longer bonds as pulse energy increases. Observed change in average Al-O bond length between P_E_ = 100 and 10 eV decreases with Al_2_O_3_/SiO_2_ concentration (shown in Tables S1, S3 and S5). Calculated Si–O average bond length across all compositions increased by less than 0.01 Å. The aforementioned densification process around silicon species, which primarily occurs from a reduction in inter-tetrahedral angle and little to no detectable change in the interatomic distance, is supported by our study. Previous studies^12,23^ investigating compression of glass melts have found changes in the O–Al–O bond angle distribution are almost exclusively associated to Al^(IV)^. Based on these results, we have determined that (1) Si species are far less susceptible to laser-induced structural modifications compared to Al, (2) distortion to the O–Al–O bond angle are dependent on temperature and pressure generated from pulse energies, and (3) increased Al_2_O_3_/SiO_2_ ratio amplifies laser modified structural distortions.

#### Intermediate range order

To better understand the effect of laser pulse interaction on intermediate range order of glass structure, we investigated the bond angle distribution between Si and Al species. Distribution statistics for Al–O–Al, Al–Al, Si–O–Si, Si–Si and Al–O–Si are provided in Tables 3 and 4 (statistics for CAS15.70, CAS20.60 and CAS25.50 are provided in the supplementary information, Tables S1–S6). Under ambient conditions, the average distance for Si–Si and Al–Al are approximately 3.10 Å^59,65^. Pulsed laser exposure increases the average distance for both; however, as previously noted in the interatomic bond angle analysis, Al species show a significantly higher capacity for distortion. An interesting observation from bond length calculations highlights a distinct elongation of Al–Al between ambient and laser-modified structure, but little to no change between P_E_ = 10 and 100 eV. Similar trends are seen in Si–Si bonding, but the magnitude of change is smaller.

**Figure 9:**
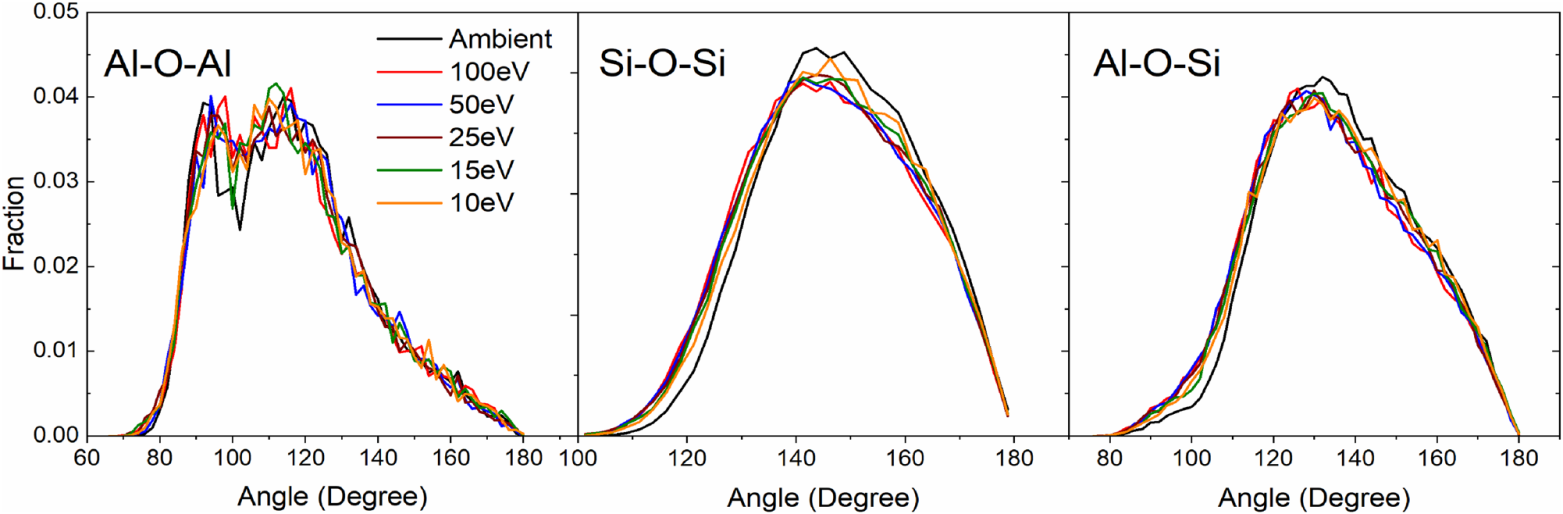
The distribution of average bond angle for Al–O–Al, Si–O–Si and Al–O–Si over the first eleven shells, relative to simulated laser pulse energy. Error is within 1%.

The average Al–O–Al, Si–O–Si and Al–O–Si bond angle in unmodified CAS10.80 are 116.1°, 148.4° and 136.8°, respectively. Based on the distributions presented in Fig. 9, the average bond angles all tend to decrease with pulse energies (resulting in concomitant increase in Al–Al and Si–Si distance). Contrary to aluminum avoidance principle, studies have shown the presence of Al–O–Al bonding in calcium aluminosilicate glasses^19,67^. In CAS10.80, average Al–O–Al angle is 116.1° under ambient conditions and 115.3° after exposure to P_E_ = 100 eV. Increasing Al concentration results in a larger distinction between the pristine and laser-damaged (P_E_ = 100 eV) average Al–O–Al bond angle (x = 0.15 117.9° and 116.3°, x = 0.2 119.3° and 117.2°, x = 0.25 119.8° and 117.6° for ambient and P_E_ = 100 eV, respectively). Heightened standard deviation to Al–O–Al distribution reflects the transition of Al four- to five-fold coordination, considering laser effects on short-range order were significantly more pronounced in Al-species. Agnello et al.^13^ have shown deconvolution of Al–O–Al bond angle distribution, in high silica content glasses, contains two distributions, (1) Al connected by a BO (Al–BO–Al) and (2) Al bonded to a triclustered oxygen (Al–TBO–Al)^13^. Reduced intensity along the distribution tail at higher angles is attributed to decreased BO and an increase around 100° indicates the formation of TBO. As the initial glass system is charge neutral, triclustered oxygen and five-coordinated Al form to compensate for the formation of NBO.

Stability of Si–O and Si–Si distance (Table 4) after laser irradiation indicates the decrease in average Si–O–Si angle is likely unrelated. Reduction is attributed to the dissociation of high-coordinated ring structures (five- and six-fold) and increase in three- and four-membered rings. This phenomenon is reported in both simulated and experimental investigations of laser modified silicate glasses. Hehlen^68^ has shown the densification of vitreous silica at 1000 K under hydrostatic pressure decreases the Si–O-Si bond angle by roughly 5.7°, equating to nearly 20% densification, as a result of breaking six-membered rings and forming three- and four-fold rings. These studies are consistent with our findings in which increasing laser pulse energy reduces average Si–O–Si bond angle, likely through the dissociation of higher-membered rings and formation of three and four-membered rings.

Al–O–Si bonding follows a similar trend as Si–O–Si bonding when exposed to laser radiation. With increasing Al concentration, the unmodified distributions are centered at 136.8°, 136.6°, 136.5° and 136.4°, respectively. Pulse energy equal to 100 eV shifts the average Al–O–Si bond angle by 2.5°, 2.7°, 2.8° and 3.0° for CAS10.80, CAS15.70, CAS20.60 and CAS25.50, respectively. The distribution shows significant dependence on temperature and pressure. Based on the changes to Al–O–Al and Si–O–Si, we attribute the shift and population increase around 100° to triclustered oxygen formation. Forming five-coordinated Al and triclustered oxygen is necessary for charge compensation of NBO. Most NBO are associated with SiO_4_ units^12^. The Ca^2+^ cation tends to be close to negatively charged species like [AlO_4_]^−^, [AlO_5_]^−^ and [NBO]^−^, therefore they are most likely to be found connecting [AlO_x_]^−^ units or along –Si–NBO units.

**Figure 10:**
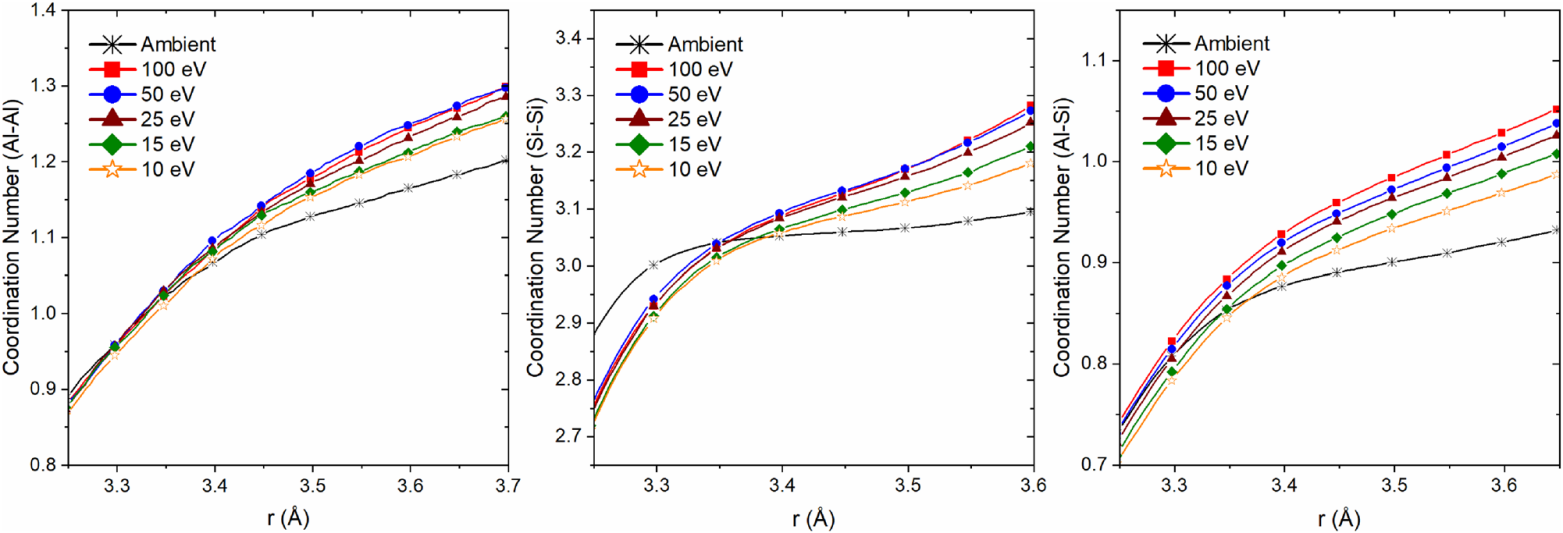
Running coordination for Al–Al, Si–Si and Al–Si after exposure to various pulse energies. Error is within 1%.

Figure 10 illustrates the coordination number between Al–Al, Si–Si and Al–Si; calculated by integrating the pair radial distribution function (PRDF). The shoulders correspond to PRDF first minimum for cutoff distance; intensity represents the average coordination number of the pair. In the case of Si–Si under ambient conditions, each SiO_x_ unit connects to approximately 3 other SiO_x_ units. Laser exposure causes a slight decrease in shoulder height, indicating a reduction in silica clusters and forming of NBO. As previously discussed, this is to be expected with increased Al^(V)^ and TBO concentrations, in order to maintain charge neutrality. Conversely, AlO_x_ clustering increases as a function of pulse energy. As previously mentioned, Al^(IV)^ species transitioning to Al^(V)^ is accompanied by an increase in TBO (predominately bonded to AlO_X_ units). Under ambient conditions Al–Si has an average coordination of 1. Similar to Al–Al, due to a slight increase in coordination as a function of pulse energy we show the pairing is susceptible to laser affects. As a result, the coordination change indicates mixing between AlO_X_ and SiO_X_ units increases with pulse energy. Based on the fact that NBO are most often found with SiO_4_ units, we can expect the modifier ion (Ca^2+^) to bridge two negatively charged species.

## Conclusion

Ultrafast pulse-laser exposure of CAS glasses was modeled using MD simulations. We have compiled a comprehensive study on the effects of pulse energy and glass composition (along the tectosilicate join) on the resolving structure. After analyzing the data, we find (1) thermally induced shockwave generation caused permanent perturbations to the average number density per shell, but does not alter localized chemistry, (2) glasses with the highest silica content incur the largest void size and densification in a single shell (up to 14%), (3) formation of fivefold Al increases with pulse energy, and (4) distortion in SiO_x_ units primarily occur to intermediate-range order(Si–O–Si, Si–Si), with minimal modification in the short-range order(O–Si–O, Si–O).

Systems with increased Al_2_O_3_ concentration have a smaller void diameter, this is attributed to longer relaxation times. Observations of the structure’s short-range order, including coordination number distribution and first nearest-neighbor bonding between Al–O and Si–O, showed the ability of Al-species to switch from four- to five-fold coordination, increasing the average Al–O bond length and reducing the O–Al–O bond angle. Shells with enriched Al^(V)^ content showed the most significant change in density. Based on statistical trends relating to Al structure, we have determined that Al coordination and the subsequent bonding environment are dependent pulse energy. Our results are consistent with the literature, confirming that structural changes of Al species have significantly higher temperature and pressure dependences compared to Si. Formation of triclustered oxygen and reduction in silicon ring size were both observed as a result of laser modification to intermediate-range order. Based on a decreased intensity in Si–Si running coordination number shoulder and increase to Al-Al and Al-Si coordination number, we postulate that the laser effects reduce clusters of SiO_X_ units via enhanced mixing of AlO_X_ species and creation of NBO to charge balance TBO and Al^(V)^.

In conclusion, we claim the laser-damaged densification process is two-fold; (1) increase in the Al^V^ concentration and (2) distortion to the silica inter-tetrahedral bonding environment (i.e. Si–O bonding distance and Si–O–Si angular bonds). Densification in glasses with higher Al_2_O_3_/SiO_2_ ratio densify less; smaller increase in Al^V^ occurs in comparison to Si-rich compositions. Understanding the complexities of laser modified glass structure, in multicomponent systems such as CAS, requires strong fundamental comprehension of both short- and medium-range order. This work serves as an important first step in creating a material map of laser-glass interaction studies. Future work should investigate structural evolution after exposure to multiple pulses and defect stability post annealing. By developing a comprehensive model, we can begin to lay the groundwork for improved structural optimization via laser-exposure, and even bridge towards laser-induced nucleation.

## Supplementary Information

Supplementary information.
