# Author Correction: Laser-induced structural modification in calcium aluminosilicate glasses using molecular dynamic simulations

**DOI:** 10.1038/s41598-021-03906-4

**Published:** 2021-12-20

**Authors:** Sean Locker, Sushmit Goyal, Matthew E. McKenzie, S. K. Sundaram, Craig Ungaro

**Affiliations:** 1 Kazuo Inamori School of Engineering, The New York State College of Ceramics, Ultrafast Materials Science and Engineering Laboratory (U-Lab), Alfred University, Alfred, NY 14802 USA; 2 Corning Incorporated, Science and Technology Division, Corning, NY 14831 USA

Correction to: *Scientific Reports* 10.1038/s41598-021-88686-7, published online 04 May 2021

In the original version of this Article, the values in Table 3 were a duplication of the values in Table 4.

The original Table 3 and accompanying legend appear below.

**Table 3:** Distribution statistics for Al–O, O–Al–O, and Al–Al under ambient and laser modified conditions in CAS10.80. These values were averaged over ten timesteps and indicate standard error.

Parameter	Ambient	10 eV	15 eV	25 eV	50 eV	100 eV
**Al–O–Al**						
Mean	148.4 ± 0.05	147.3 ± 0.06	146.8 ± 0.06	146.5 ± 0.06	146.2 ± 0.06	146.0 ± 0.06
Median	148.2	147.2	146.7	146.4	146.0	145.8
SD	13.9	14.5	14.8	14.9	15.0	15.0
**Al–O–Si**						
Mean	109.3 ± 0.01	109.2 ± 0.02	109.2 ± 0.02	109.2 ± .02	109.2 ± .02	109.2 ± 0.02
Median	108.9	108.6	108.6	108.5	108.4	108.4
SD	6.70	8.30	8.54	8.82	9.01	9.05
**O–Al–O**						
Mean	1.61 ± 0.00	1.62 ± 0.00	1.62 ± 0.00	1.62 ± 0.00	1.62 ± 0.00	1.62 ± 0.00
Median	1.61	1.62	1.62	1.62	1.62	1.62
SD	0.05	0.07	0.07	0.07	0.07	0.07
**d(Al–O)**						
Mean	3.098 ± 0.000	3.106 ± 0.001	3.105 ± 0.001	3.105 ± 0.001	3.106 ± 0.001	3.108 ± 0.001
Median	3.102	3.111	3.108	3.108	3.106	3.106
SD	0.110	0.130	0.134	0.135	0.136	0.138
**d(Al–Al)**						
Mean	148.4 ± 0.05	147.3 ± 0.06	146.8 ± 0.06	146.5 ± 0.06	146.2 ± 0.06	146.0 ± 0.06
Median	148.2	147.2	146.7	146.4	146.0	145.8
SD	13.9	14.5	14.8	14.9	15.0	15.0

In addition, the legend of Table 4,

“Distribution statistics for Al–O, O–Al–O, and Al–Al under ambient and laser modified conditions in CAS10.80.”

now reads:

“Distribution statistics for Si-O, O-Si-O, and Si-Si under ambient and laser modified conditions in CAS10.80.”

The original Article has been corrected.

