# Supplementary material for: Laser-induced structural modification in calcium aluminosilicate glasses using molecular dynamic simulations
Source: Sci Rep. 2021 May 4;11:9519. doi: 10.1038/s41598-021-88686-7 (PMC8096823; doi:10.1038/s41598-021-88686-7)
Supplement: Supplementary file 1 — Supplementary information. [file 41598_2021_88686_MOESM1_ESM.pdf]

Laser-induced structural modification in calcium aluminosilicate glasses using molecular dynamic simulations

Sean Locker[1*], Sushmit Goyal[2], Matthew E. McKenzie[2], SK Sundaram[1], Craig Ungaro[2]

[1]Kazuo Inamori School of Engineering, The New York State College of Ceramics, Ultrafast Materials Science and Engineering Laboratory (U-Lab), Alfred University, Alfred, New York 14802, USA
[2]Corning Incorporated, Science and Technology Division, Corning, NY 14831, USA

Supplementary Information

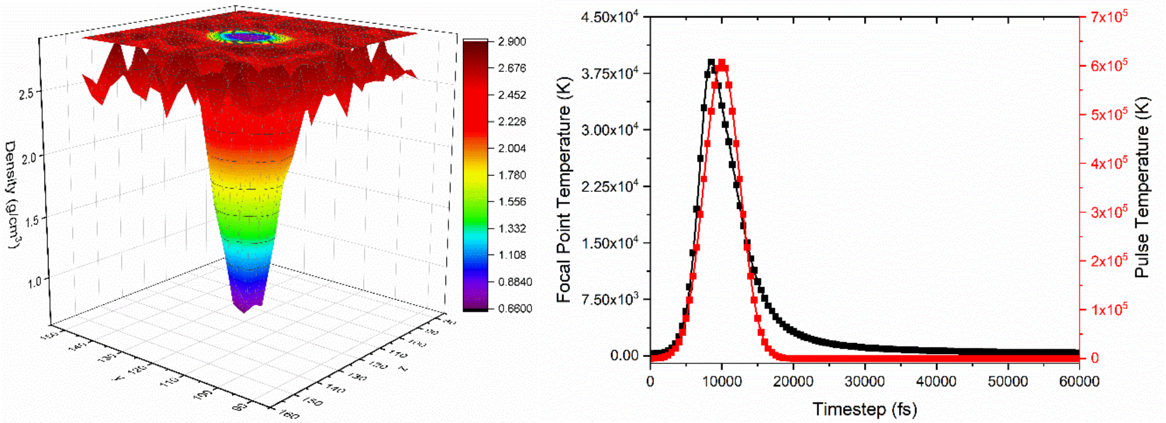

Figure S1. Calcium aluminosilicate glass density around the focal diameter of simulated laser pulse at the peak intensity (left), Temperature profile of the pulse (P_E = 100 eV) (red) and the focal volume (black)(right).

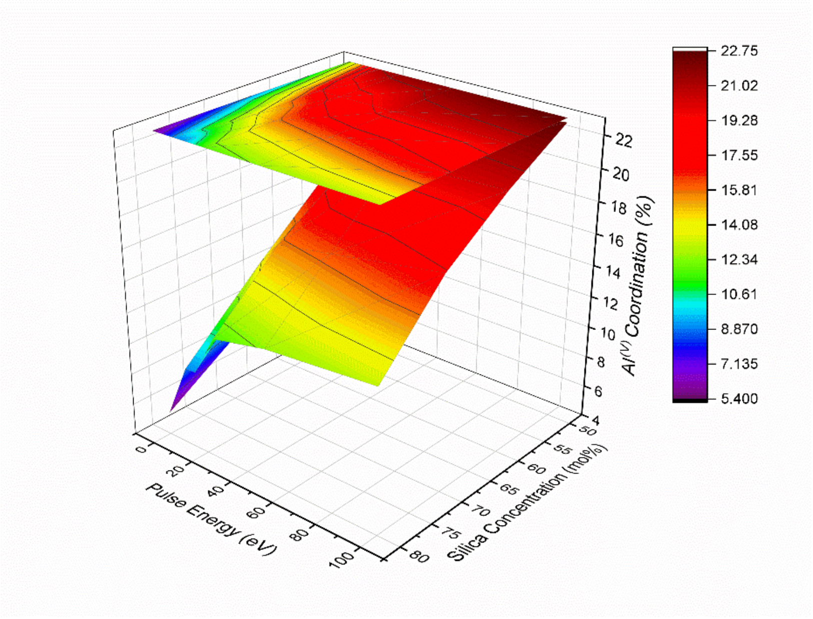

Figure S2. Five-coordinated Al for all simulated CAS systems as a function of silica concentration and pulse energy.

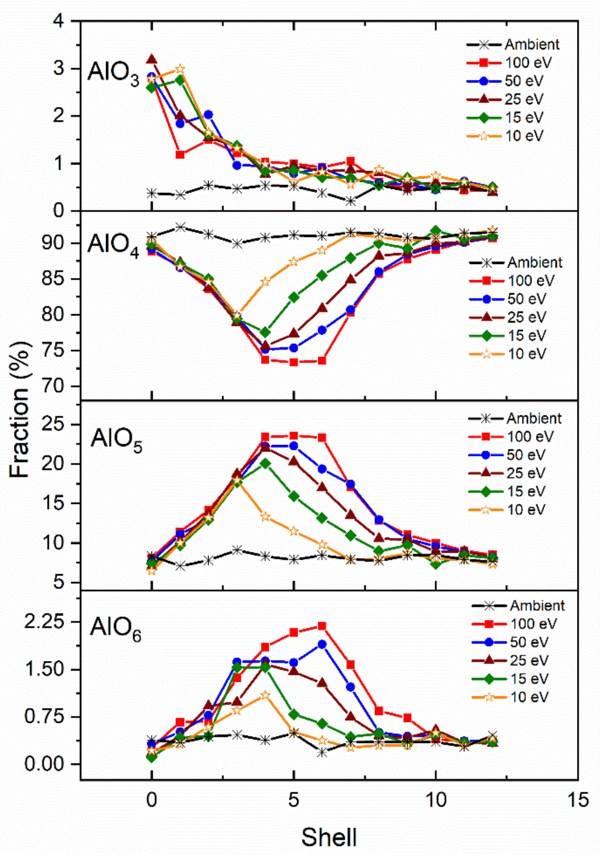

Figure S3. Distribution of CAS15.70 AlO_x units (at x = 3, 4, 5 and 6) as a function of shell number. Shell zero represents the combine statistics within the focal volume (shells one through five).

Table S1. Distribution statistics for Al-O, Al-Al and Al-O-Si bonding under ambient and laser modified conditions in CAS15.70

Parameter		Ambient	10 eV	15 eV	25 eV	50 eV	100 eV
Al-O-Al	Mean	117.9 ± 0.16	117.4 ± 0.16	116.9 ± 0.16	116.6 ± 0.16	116.5 ± 0.17	116.3 ± 0.16
	Median	116.6	115.8	115.1	114.8	114.7	114.6
	Std. dev.	20.1	20.3	20.3	20.2	20.3	20.3
Al-O-Si	Mean	136.6 ± 0.08	135.4 ± 0.08	134.9 ± 0.08	134.5 ± 0.08	134.2 ± 0.08	133.9 ± 0.08
	Median	135.3	134.2	133.7	133.3	133.0	132.9
	Std. dev.	17.6	18.3	18.4	18.6	18.5	18.6
O-Al-O	Mean	108.7 ± 0.04	108.4 ± 0.05	108.3 ± 0.05	108.2 ± 0.06	108.2 ± 0.06	108.1 ± 0.06
	Median	107.2	106.2	105.8	105.5	105.3	105.2
	Std. dev.	14.3	16.6	17.4	17.9	18.1	18.4
d(Al-O)	Mean	1.769 ± 0.000	1.790 ± 0.000	1.794 ± 0.000	1.796 ± 0.000	1.797 ± 0.001	1.798 ± 0.001
	Median	1.750	1.768	1.771	1.772	1.773	1.774
	Std. dev.	0.099	0.121	0.123	0.124	0.124	0.125
d(Al-Al)	Mean	3.105 ± 0.002	3.132 ± 0.002	3.133 ± 0.002	3.133 ± 0.002	3.137 ± 0.002	3.134 ± 0.002
	Median	3.098	3.128	3.128	3.129	3.132	3.135
	Std dev.	0.263	0.270	0.275	0.275	0.279	0.278

Table S2. Distribution statistics for Si-O, Si-Si and -O-Si bonding under ambient and laser modified conditions in CAS15.70

Parameter		Ambient	10 eV	15 eV	25 eV	50 eV	100 eV
Si-O-Si	Mean	148.0 ± 0.06	146.8 ± 0.07	146.3 ± 0.07	146.0 ± 0.07	146.0 ± 0.07	145.9 ± 0.07
	Median	147.8	146.8	146.2	145.8	145.8	145.6
	Std. dev.	14.1	14.7	14.9	15.0	15.1	15.1
O-Si-O	Mean	109.3 ± .01	109.2 ± .02	109.2 ± .02	109.2 ± .02	109.2 ± .02	109.2 ± .02
	Median	108.8	108.6	108.5	108.5	108.5	108.4
	Std. dev.	6.68	8.38	8.68	8.84	8.92	9.02
d(Si-O)	Mean	1.611 ± 0.000	1.621 ± 0.000	1.621 ± 0.000	1.621 ± 0.000	1.622 ± 0.000	1.622 ± 0.00
	Median	1.607	1.614	1.614	1.614	1.614	1.614
	Std. dev.	0.054	0.074	0.074	0.074	0.074	0.074
d(Si-Si)	Mean	3.096 ± 0.000	3.111 ± 0.001	3.109 ± 0.001	3.108 ± 0.001	3.110 ± 0.000	3.109 ± 0.001
	Median	3.102	3.111	3.111	3.109	3.110	3.109
	Std dev.	0.111	0.133	0.135	0.137	0.138	0.139

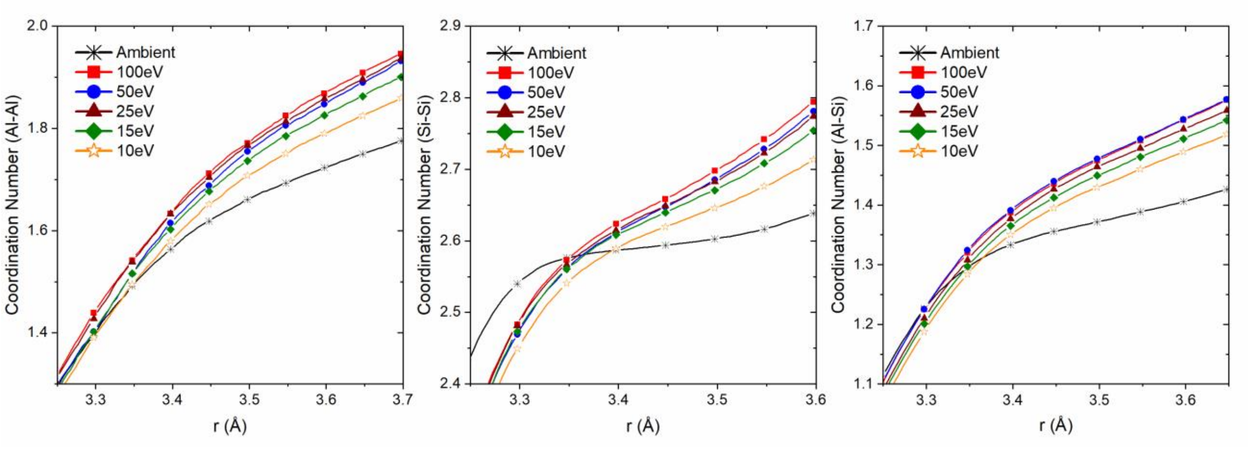

Figure S4. Running coordination for Al-Al, Si-Si and Al-Si (CAS15.70) after exposure to various pulse energies

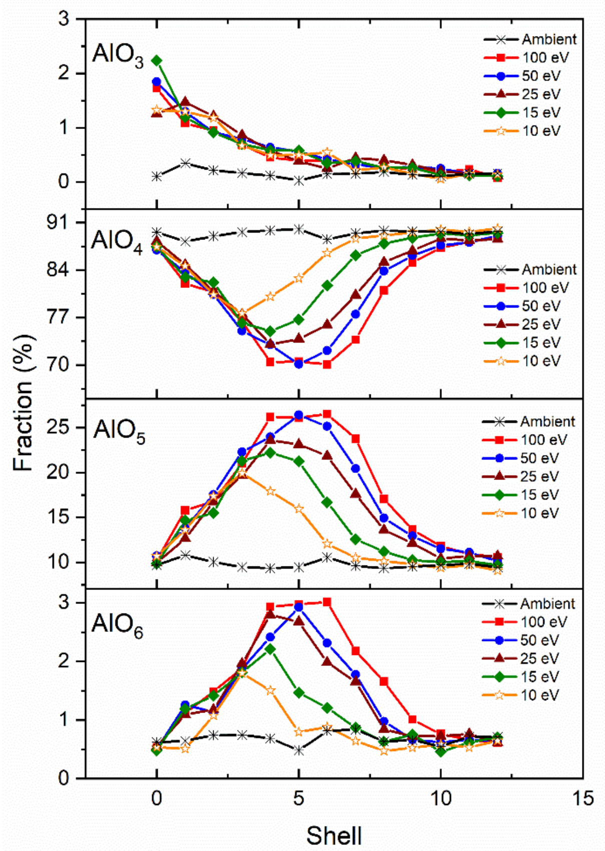

Figure S5. Distribution of CAS20.60 AlO_x units (at x = 3, 4, 5 and 6) as a function of shell number. Shell zero represents the combine statistics within the focal volume (shells one through five).

Table S3. Distribution statistics for Al-O, Al-Al and Al-O-Si bonding under ambient and laser modified conditions in CAS20.60

Parameter		Ambient	10 eV	15 eV	25 eV	50 eV	100 eV
Al-O-Al	Mean	119.3 ± 0.12	118.1 ± 0.12	117.5 ± 0.12	117.2 ± 0.12	117.2 ± 0.12	117.2 ± 0.12
	Median	118.1	116.9	116.3	115.9	115.8	115.9
	Std. dev.	19.2	19.8	20.0	19.9	20.13	20.0
Al-O-Si	Mean	136.5 ± 0.07	135.1 ± 0.07	134.4 ± 0.07	134.1 ± 0.07	133.7 ± 0.07	133.7 ± 0.08
	Median	135.2	134.0	133.2	132.9	132.4	132.4
	Std. dev.	17.1	17.9	18.0	18.1	18.2	18.2
O-Al-O	Mean	108.6 ± 0.04	108.3 ± 0.05	108.1 ± 0.05	108.1 ± 0.05	108.0 ± 0.05	108.0 ± 0.05
	Median	107.1	105.8	105.4	105.1	104.9	104.8
	Std. dev.	14.7	17.5	18.3	18.6	18.9	19.1
d(Al-O)	Mean	1.769 ± 0.00	1.795 ± 0.000	1.798 ± 0.000	1.799 ± 0.000	1.801 ± 0.000	1.801 ± 0.000
	Median	1.750	1.772	1.775	1.776	1.777	1.777
	Std. dev.	0.098	0.123	0.125	0.125	0.126	0.126
d(Al-Al)	Mean	3.110 ± 0.002	3.141 ± 0.002	3.142 ± 0.002	3.139 ± 0.002	3.139 ± 0.002	3.139 ± 0.002
	Median	3.103	3.136	3.139	3.134	3.137	3.137
	Std dev.	0.252	0.269	0.273	0.275	0.274	0.271

Table S4. Distribution statistics for Si-O, Si-Si and -O-Si bonding under ambient and laser modified conditions in CAS20.60

Parameter		Ambient	10 eV	15 eV	25 eV	50 eV	100 eV
Si-O-Si	Mean	148.0 ± 0.08	146.6 ± 0.08	146.1 ± 0.08	145.8 ± 0.08	145.7 ± 0.08	145.4 ± 0.08
	Median	148.1	146.4	145.8	145.4	145.5	145.4
	Std. dev.	14.1	14.8	15.0	15.0	15.2	15.1
O-Si-O	Mean	109.3 ± 0.01	109.2 ± 0.02	109.2 ± 0.02	109.2 ± 0.02	109.2 ± 0.02	109.2 ± 0.02
	Median	108.9	108.6	108.6	108.6	108.5	108.5
	Std. dev.	6.45	8.34	8.68	8.82	8.93	8.97
d(Si-O)	Mean	1.610 ± 0.000	1.621 ± 0.000	1.621 ± 0.000	1.621 ± 0.000	1.621 ± 0.000	1.621 ± 0.000
	Median	1.606	1.613	1.613	1.613	1.613	1.613
	Std. dev.	0.053	0.074	0.075	0.075	0.075	0.074
d(Si-Si)	Mean	3.100 ± 0.000	3.116 ± 0.001	3.114 ± 0.001	3.112 ± 0.001	3.112 ± 0.001	3.111 ± 0.001
	Median	3.106	3.117	3.114	3.111	3.112	3.111
	Std dev.	0.110	0.134	0.137	0.138	0.139	0.140

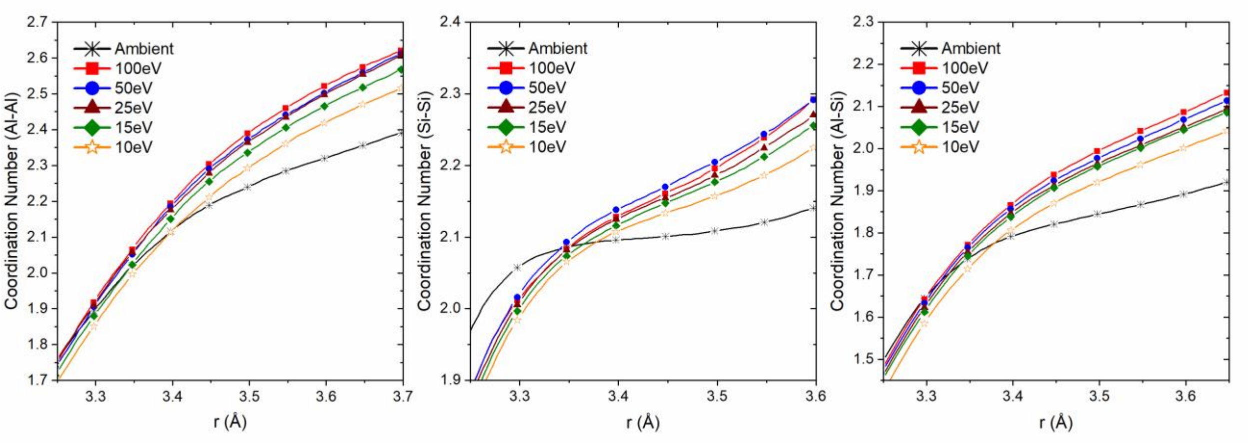

Figure S6. Running coordination for Al-Al, Si-Si and Al-Si (CAS20.60) after exposure to various pulse energies

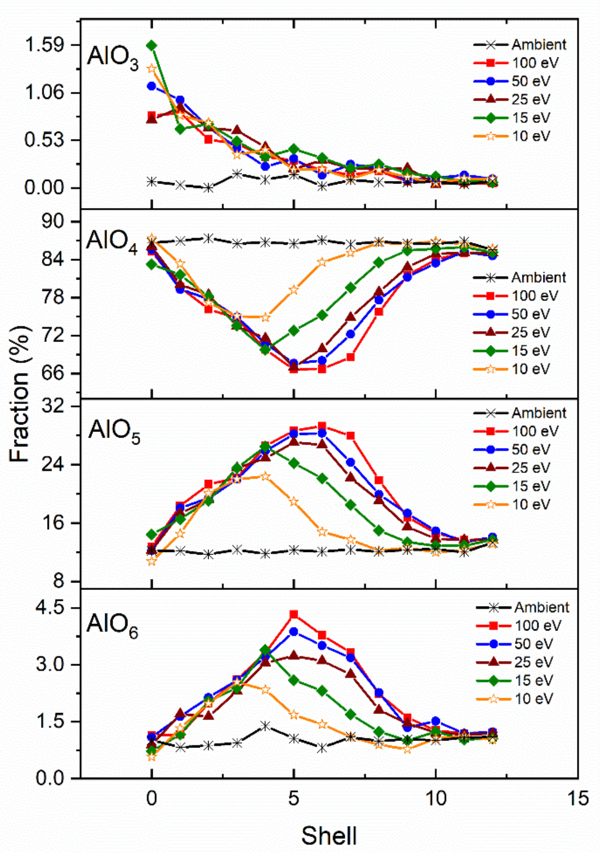

Figure S7. Distribution of CAS25.50 units (at x = 3, 4, 5 and 6) as a function of shell number. Shell zero represents the combine statistics within the focal volume (shells one through five).

Table S5. Distribution statistics for Al-O, Al-Al, Al-O-Si bonding under ambient and laser modified conditions in CAS25.50

Parameter		Ambient	10 eV	15 eV	25 eV	50 eV	100 eV
Al-O-Al	Mean	119.8 ± 0.09	118.4 ± 0.10	117.8 ± 0.09	117.5 ± 0.10	116.5 ± 0.11	117.6 ± 0.10
	Median	118.9	117.4	116.8	116.5	116.2	116.4
	Std. dev.	18.8	19.6	19.9	19.9	19.8	20.0
Al-O-Si	Mean	136.4 ± 0.07	134.8 ± 0.07	134.0 ± 0.07	133.7 ± 0.07	132.2 ± 0.09	133.4 ± 0.07
	Median	134.7	133.5	132.6	132.3	131.8	132.1
	Std. dev.	16.8	17.6	17.6	17.7	20.4	17.8
O-Al-O	Mean	108.5 ± 0.04	108.1 ± 0.04	108.0 ± 0.04	107.9 ± 0.05	107.6 ± 0.05	107.6 ± 0.05
	Median	106.7	105.4	104.9	104.7	104.2	104.1
	Std. dev.	15.6	18.3	19.1	19.3	20.3	20.4
d(Al-O)	Mean	1.772 ± 0.000	1.799 ± 0.000	1.802 ± 0.000	1.803 ± 0.000	1.804 ± 0.000	1.805 ± 0.000
	Median	1.753	1.776	1.778	1.779	1.780	1.780
	Std. dev.	0.100	0.125	0.126	0.127	0.123	0.124
d(Al-Al)	Mean	3.112 ± 0.001	3.146 ± 0.001	3.146 ± 0.001	3.145 ± 0.001	3.145 ± 0.001	3.144 ± 0.001
	Median	3.110	3.141	3.140	3.140	3.140	3.139
	Std dev.	0.251	0.268	0.273	0.2274	0.274	0.272

Table S6. Distribution statistics for Si-O, Si-Si and -O-Si bonding under ambient and laser modified conditions in CAS25.50

Parameter		Ambient	10 eV	15 eV	25 eV	50 eV	100 eV
Si-O-Si	Mean	147.7 ± 0.09	146.2 ± 0.10	145.6 ± 0.10	145.4 ± 0.10	142.3 ± 0.10	145.2 ± 0.10
	Median	147.4	146.1	145.2	145.0	143.5	144.8
	Std. dev.	14.1	14.9	15.1	15.0	20.1	15.2
O-Si-O	Mean	109.3 ± 0.02	109.2 ± 0.02	109.2 ± 0.02	109.2 ± 0.02	109.2 ± .02	109.2 ± .02
	Median	108.9	108.6	108.6	108.5	108.5	108.5
	Std. dev.	6.45	8.40	8.61	8.67	8.74	8.78
d(Si-O)	Mean	1.609 ± 0.000	1.620 ± 0.000	1.620 ± 0.000	1.620 ± 0.000	1.620 ± 0.000	1.620 ± 0.000
	Median	1.605	1.613	1.612	1.612	1.612	1.612
	Std. dev.	0.053	0.075	0.075	0.074	0.075	0.074
d(Si-Si)	Mean	3.101 ± 0.001	3.117 ± 0.001	3.117 ± 0.001	3.115 ± 0.001	3.114 ± 0.001	3.115 ± 0.001
	Median	3.107	3.118	3.116	3.115	3.112	3.114
	Std dev.	0.111	0.136	0.139	0.140	0.141	0.140

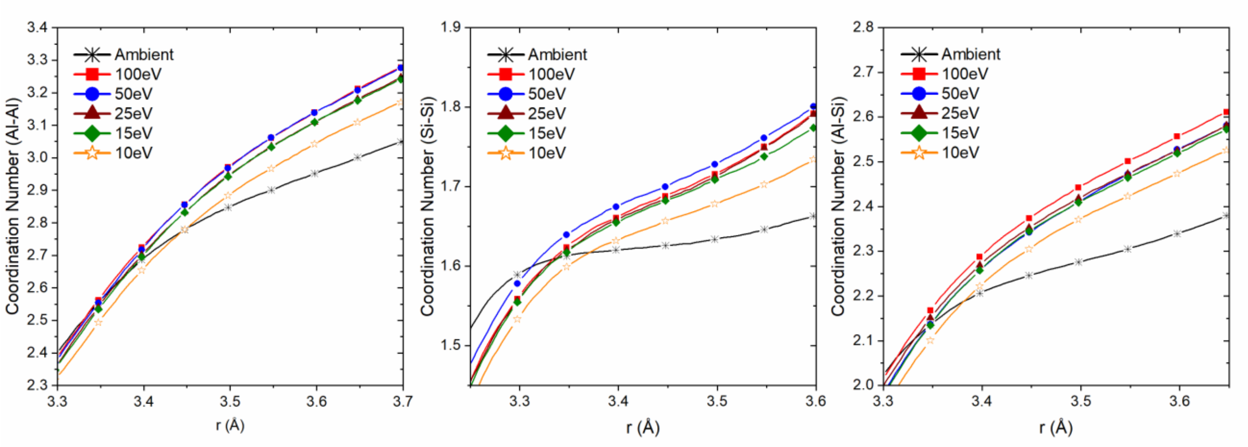

Figure S8. Running coordination for Al-Al, Si-Si and Al-Si (CAS25.50) after exposure to various pulse energies

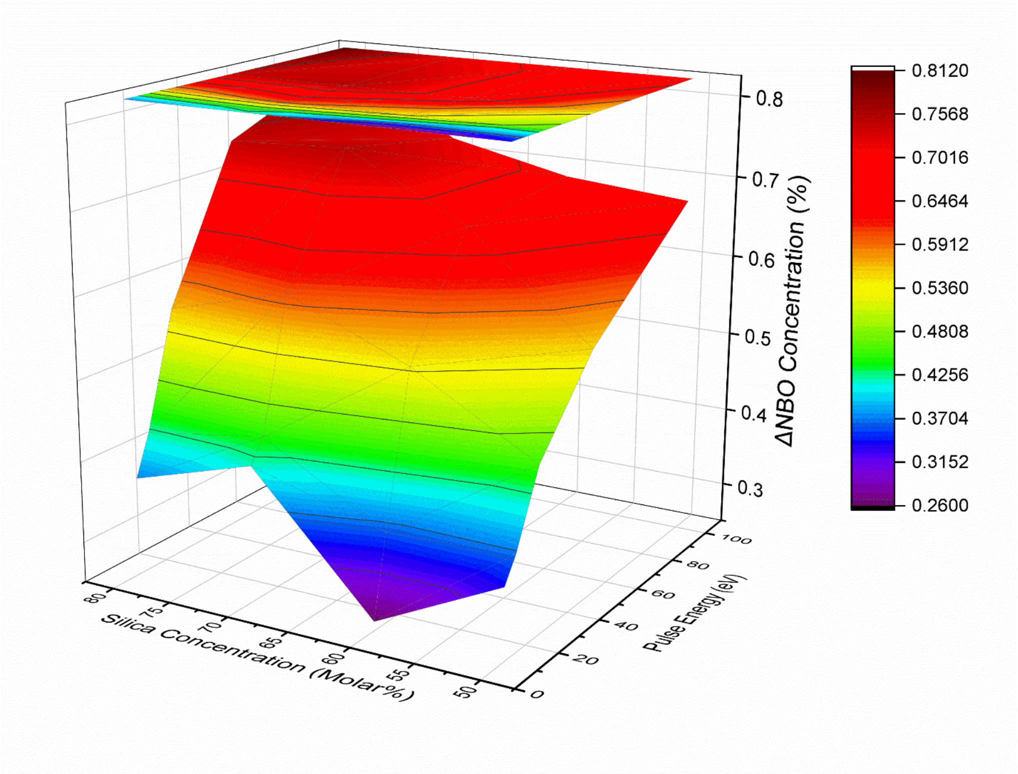

Figure S9. Fractional change in non-bridging oxygen (NBO) content as a function of silica concentration and pulse energy

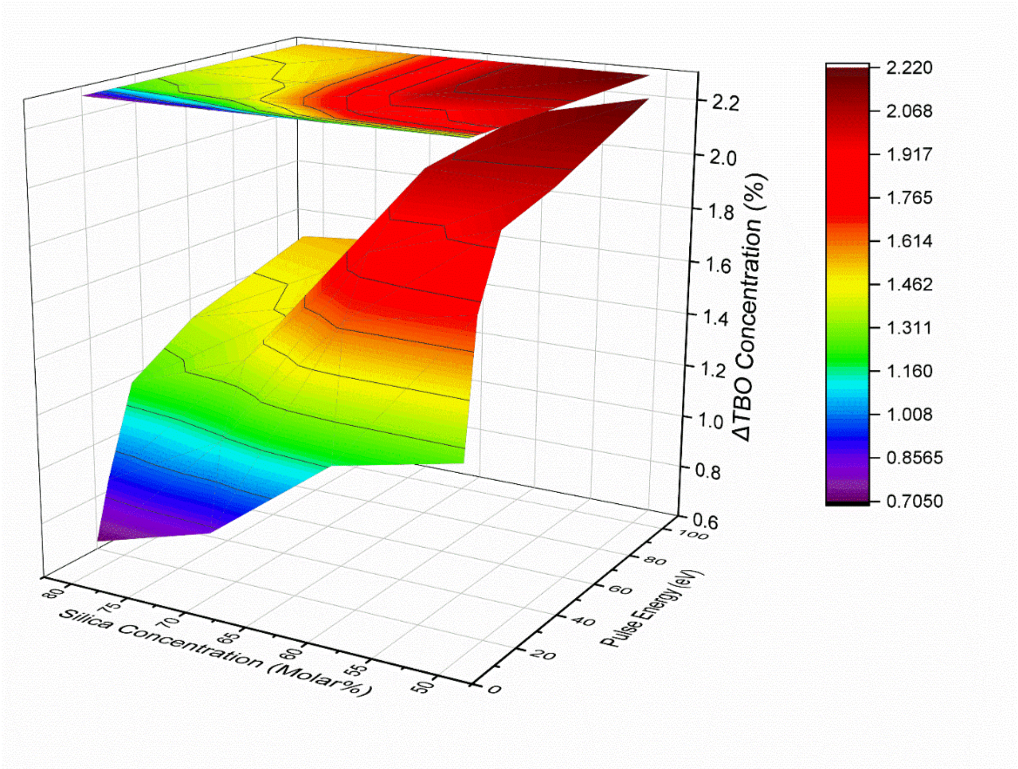

Figure S10. Fractional change in triclustered oxygen (TBO) content as a function of silica concentration and pulse energy.

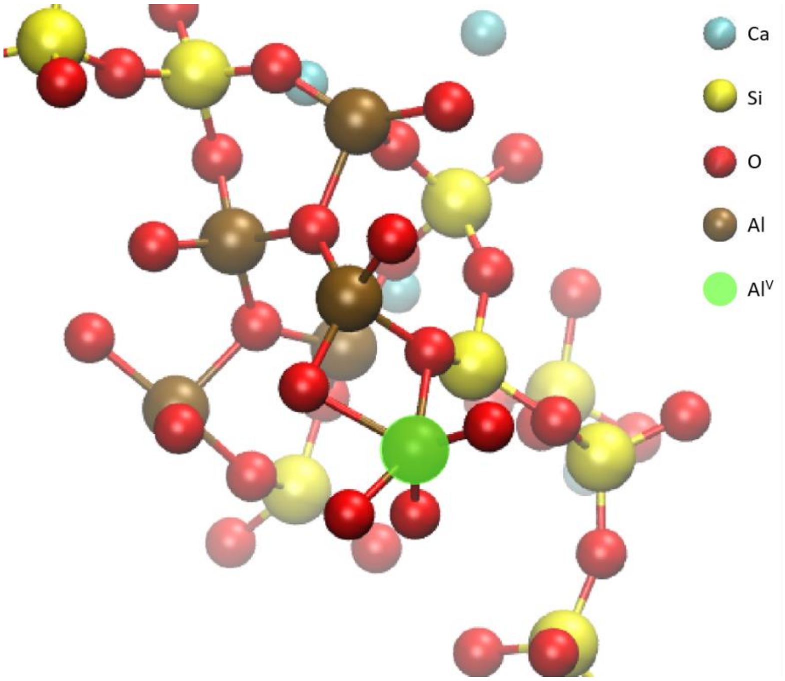

Figure S11. Al^{V} in CAS10.80 (VMD, 1.9.4, http://www.ks.uiuc.edu/Research/vmd/)

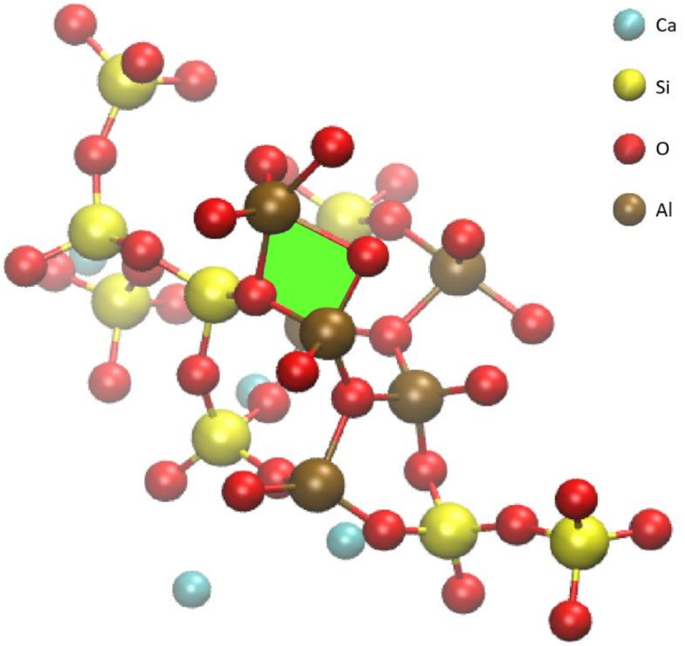

Figure S12. 2-membered ring in CAS10.80 between Al^{IV} and A^{V} (VMD, 1.9.4, http://www.ks.uiuc.edu/Research/vmd/)

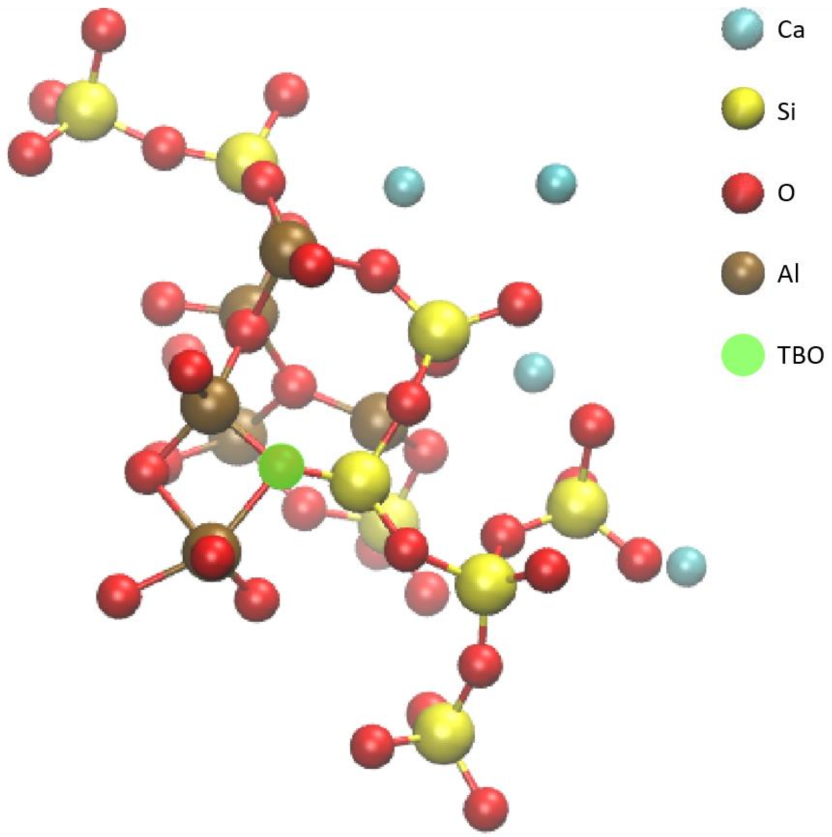

Figure S13. TBO formed in CAS10.80 between Al- and Si-tetrahedra and Al^{V} (VMD, 1.9.4, http://www.ks.uiuc.edu/Research/vmd/)
